# PTP4A3 Is a Prognostic Biomarker Correlated With Immune Infiltrates in Papillary Renal Cell Carcinoma

**DOI:** 10.3389/fimmu.2021.717688

**Published:** 2021-09-23

**Authors:** Qian Song, Yutian Zheng, Junzhou Wu, Sheng Wang, Lin Meng, Qian Yao, Zhongwu Li, Shenyi Lian

**Affiliations:** ^1^ Department of Clinical Laboratory, The Cancer Hospital of the University of Chinese Academy of Sciences (Zhejiang Cancer Hospital); Institute of Basic Medicine and Cancer (IBMC), Chinese Academy of Sciences, Hangzhou, China; ^2^ Key Laboratory of Carcinogenesis and Translational Research (Ministry of Education/Beijing), Department of Biochemistry and Molecular Biology, Peking University Cancer Hospital & Institute, Beijing, China; ^3^ Cancer Research Institute, The Cancer Hospital of the University of Chinese Academy of Sciences (Zhejiang Cancer Hospital); Institute of Basic Medicine and Cancer (IBMC), Chinese Academy of Sciences, Hangzhou, China; ^4^ Key laboratory of Carcinogenesis and Translational Research (Ministry of Education/Beijing), Department of Pathology, Peking University Cancer Hospital & Institute, Beijing, China

**Keywords:** renal cancer, PTP4A3, prognosis, tumor infiltration, biomarker

## Abstract

PTP4A3 plays an important role in the tumorigenesis and metastasis of multiple tumors, but its prognostic role in renal cancer is not well understood. We utilized the Oncomine and Tumor Immunoassay Resource databases to examine the differential expression of PTP4A3 in tumor tissues and normal tissues in breast, urinary tract, gastrointestinal tract and skin. Using the GEPIA and PrognoScan databases, the independent prognostic role of PTP4A3 was confirmed in clear cell renal cell cancer and papillary renal cell cancer. Expression of PTP4A3 were obviously higher in tumor tissue compare with normal tissues (*P*=0.028). We haven’t found the associations of PTP4A3 and clinicopathological features in our IHC cohort. Ectopic expression of PTP4A3 promotes proliferation, migration and invasion and increased the mRNA level of TGFB1 in RCC cell lines. Immunohistochemical staining indicated that the expression of PTP4A3 associates with CD3+ (*P* =0.037)/CD8+ (*P* =0.037) intratumor TILs, not with invasive margins in renal cancer. Comprehensive analysis of immune infiltration in the TIMER database correlated PTP4A3 expression with the infiltration of B cells, CD8+ T cells, CD4+ T cells and neutrophils in both clear cell renal cell carcinoma and papillary renal cell carcinoma. PTP4A3 expression was associated with the infiltration of dendritic cells in papillary renal cell carcinoma. We further confirmed that the infiltration of B cells and CD8+ T cells was associated with poor prognosis in papillary renal cell carcinoma patients, consistent with the prognostic role of PTP4A3 in papillary renal cell carcinoma. PTP4A3 expression correlated genes involved in B cells, monocytes, M1 macrophages, Th2 and Treg cells in papillary renal cell carcinoma. These results suggest PTP4A3 as a prognostic factor with a role in regulating immune cell infiltration in papillary renal cell carcinoma.

## Introduction

Renal cell carcinoma (RCC) is a common malignant disease expected to account for approximately 76,080 cases and 13,780 deaths in 2021 in the United States (1). RCC is divided into three major histologic subtypes: clear cell RCC (ccRCC or KIRC), papillary cell RCC (pRCC or KIRP), and chromophobe RCC (chRCC). According to The Cancer Genome Atlas (TCGA), a comprehensive database of cancer genomic profiles, these three major RCC subtypes have distinctive genetic alterations including genomic mutations, methylation status, RNA expression signatures, and immune signatures (2–4). At diagnosis, 30% of RCC patients were confirmed as distant metastatic (5). While localized RCC could be cured by surgical resection, about 40% of surgical patients eventually experienced recurrence. Recently, adjuvant therapy, and especially immune therapy, has received significant attention as an option for treating metastatic RCC (mRCC). Immune therapy combined with a tyrosine kinase inhibitor; bevacizumab, a monoclonal antibody against vascular endothelial growth factor; or ipilimumab, a monoclonal antibody against cytotoxic T-lymphocyte antigen-4 are now considered optimal treatment strategies for mRCC. The biomarkers Programmed Cell Death Protein 1 (PD1) and Programmed Death-Ligand 1 (PD-L1) exhibited higher expression in ccRCC than in chRCC or pRCC, and high expression was correlated with decreased survival in all RCC patients (*P*=0.02) (6, 7). Immune cell genes signatures, including those of B cells, dendritic cells, CD68, CD8, Treg cells, Th1/2 and immunoglobin G, were examined in ccRCC, pRCC and chRCC. In general, expression was higher in ccRCC than pRCC and chRCC, except for Th17, IL-8, and CD56^bright^natural killer (NK) cell signatures. Th2 gene signatures were universally correlated with poor overall survival (OS) in all major histological subtypes (2). IL-8 inflammatory pathway-related genes and CD56^bright^NK cell genes had higher expression in pRCC than in ccRCC and chRCC, whereas IL-17 producing T helper cells (T_H_17 cells) had lower expression in pRCC than in ccRCC and chRCC. T_H_17 gene signatures positively correlated with OS in ccRCC and chRCC (*P*=0.0021 and *P*=0.0362, respectively). CpG island methylator phenotype-associated RCC had increased expression of T_H_2 cells and Treg cells compared with pRCC and chRCC. Therefore, immune biomarkers for different subtypes of RCC need to be explored.

Protein Tyrosine Phosphatase 4A3 (PTP4A3), also known as phosphatase of regenerating liver 3 (PRL-3), is a protein tyrosine phosphatase distributed in the cell nucleus, plasma membrane and endosome (8). It is negatively correlated with survival in colon cancer (9), breast cancer (10, 11), gastric cancer (12–14), ovarian cancer (15, 16) and acute myelogenous leukemia (AML) (17). PTP4A3 promotes the proliferation, migration, and invasion of tumor cells by activating the Rho family and matrix metalloproteinase-2. PTP4A3 has been shown to continuously activate the PI3K-AKT-ERK pathway by negatively regulating Src kinase and PTEN (18, 19). PTP4A3 is known to promote epithelial mesenchymal transition by KCNN4 channels (20), stimulate G2/M cell cycle arrest by ubiquitinating AURKA and dephosphorylating FZR1 (21), increase IL-1 alpha secretion through the NF-κB and JAK2-STAT pathway (22). Extensive data supports the deprotection role of PTP4A3 in tumorigenesis and telomere maintenance (23, 24). Studies in PTP4A3 transgenic mice have shown that high expression of PTP4A3 promotes colitis-related colon cancer, shortens telomeres, and increases expression of H3K9Me, a hallmark of genomic instability (24, 25). PTP4A3 restricts transcription in melanoma through the DDX21-MITF axis (26).

Due to the important role of PTP4A3 in cancers, a PRL-zumab was generated by the Zeng group and was shown to specifically inhibit PTP4A3-positive liver cancer cell *in vivo*, and recruit B cells, NK cells and macrophages to the PTP4A3-positive tumor microenvironment (27, 28). A DNA vaccine targeting PTP4A3 triggered high expression of interferon-γ and TNF-α in breast cancer through the CTL and T helper type 1 cells immune response and also stimulated the accumulation of PTP4A3 antibody in immunized mice (29). IL-6/8 secreted by tumor-associated macrophages (TAMs) facilitated the metastasis of colon cancer in a PTP4A3-KCNN4-dependent manner (30). Few reports have examined the role of PTP4A3 in kidney cancer, and this research has mostly focused on the correlation of clinicopathological characteristics and prognosis. The mechanism of how PTP4A3 regulates immune cells and its impact on renal cancer prognosis remains unclear.

Herein, we developed a comprehensive analysis across multiple databases to elucidate the role of PTP4A3 in ccRCC and pRCC. In addition, we analyzed the relationship of PTP4A3 and immune cell tumor infiltration (TIMER) (31, 32). This study offers new insights into the function of PTP4A3 in renal cancer and proposes a mechanism for PTP4A3 regulating tumor infiltration of immune cells.

## Materials and Methods

### Oncomine Database Analysis

The Oncomine database comprises a wide range of gene expression datasets covering various cancer types. PTP4A3 expression in diverse tumor types was investigated using this database (https://www.oncomine.org/resource/login.html) (33) with the screening thresholds set as: P-value of 0.001, fold change of 1.5 and gene rank of top 5%.

### PrognoScan Database Analysis

The PrognoScan database complies accessible tumor microarray datasets, facilitating the correlation of gene expression and survival including OS and disease-free survival (DFS). We evaluated the relationship between PTP4A3 expression and prognosis in diverse tumor types using the PrognoScan database (http://www.abren.net/PrognoScan/) (34) with a threshold P-value of 0.05.

### Cell Culture and Antibody

Kidney clear carcinoma cell lines 786-O and Caki-2 were obtained from the Cell Resource Center, Peking Union Medical College (which is the headquarters of National Infrastructure of Cell Line Resource, NSTI. 786-O cell were cultured in RPMI-1640 (Invitrogen, Carlsbad, CA, USA) containing 10% fetal bovine serum. Caki-2 cell were cultured in McCoys’5A (Gibco, USA) containing 10% fetal bovine serum. Anti-Myc tag (AB 103) was from TianGen Biotech (Beijing, China). Anti-GAPDH (10494-1-AP) was from Proteintech Group (Chicago, IL, USA).

### Lentivirus Infection and Western Blot

To stable expression of ectopic PTP4A3 in renal cancer cell, 786-O and Caki-2 cells were infected with 50 MOI control or myc-PTP4A3 expression lentivirus for 72 hours. Cells were directly homogenized in 2 × loading buffer to get the whole cell extracts. Protein samples were separated by the SDS-polyacrylamide gel electrophoresis and transferred onto the nitrocellulose membranes, and blocked in 5% milk in PBS buffer for 1 hour. The lentivirus infection were detected by the myc-tag antibody (1μg/ml) at 4°C overnight and washed in 0.1% Tween in PBS for 3 times. After incubating with HRP-conjugated anti-mouse secondary antibody, the protein bands were visualized by the enhanced chemiluminescence detection system (Thermo Fisher Scientific, Pittsburgh, PA, USA).

### Cell Proliferation Migration and Invasion

Cell Counting Kit (CCK)-8 (C0037, Beyotime, Shanghai, China) were used for cell proliferation experiments. 3 ×10^4^ cells of 786-O vector, 786-O-myc-PTP4A3, Caki-2 vector and Caki-2-myc-PTP4A3 cells were re-suspended in 100 μl complete medium, and seed in the 96-well plates in triplicate. At 0,24,48,72,96 hours, discarded the medium and added 100μl fresh medium containing 10 μl CCk-8 to each well, After 2 hours incubation at 37°C, the spectrophotometer (Thermo Fisher Scientific, Pittsburgh, PA, USA)was used to measure the absorbance of each well at OD450nm.

200 μl re-suspended 786-O vector, 786-O-myc-PTP4A3, Caki-2 vector and Caki-2-myc-PTP4A3 cells were seeded on the upper chamber of each transwell (Becton Dickinson, San Jose, CA, USA), 2×10^5^/ml for migration and 10×10^5^/ml for invasion, 800 μl medium containing 10% FBS added to the lower chamber. Cells were cultured at 37°C 24 hour for cell migration, 48 hours for cell invasions. The cells on the upper chambers were fixed in cold methanol and stained in 0.1% crystal violet for 30 mins at room temperature. The cells penetrated to the upper chamber were counted in at least 6 randomly selected field under microscope.

### Quantitative Real-Time PCR

Total RNA was extracted from cells with Trizol reagent (Invitrogen, Carlsbad, CA, USA) based on the manufacturer’s instruction. 1 μg of RNA was utilized to synthesize cDNA with GoScriptTM Reverse Transcription system (A5001, Promega). qRT-PCR was conducted with SYBR Green PCR master mix reagents (TOYOBO) and a StepOne Real-time PCR system (Applied Biosystems). Expression data of indicative gene was normalized to that of GAPDH. Primers used are listed in **Supplementary Table 4**.

### Immunohistochemical Analysis

The kidney cancer tissue array (DC-Kid11051) were bought from Avilabio company (Shanxi). DC-Kid11051 were including 33 KIRC, 9 KIRP, 5 KICH and 6 normal kidney tissues. The sections were deparaffinized in xylene and hydrate in alcohol. Endogenous peroxidase activity was then blocked by incubation in 3% hydrogen peroxide–methanol for 10 min. For the antigen recovery, the section was heated in a citrate buffer (pH 6.0) for 15mins. Incubation with PTP4A3 antibody (11) (5μg/ml, monoclonal antibody 3B6 previous validated), CD3 antibody ((Monoclonal Rabbit Anti-Human CD3, Clone 2GV6, prediluted, Roche) and CD8 antibody (Monoclonal Mouse Anti-Human CD8, Clone C8/144B, Dako) at 4°C overnight. EnVision^+^ TM (Dako, Carpinteria, CA, USA) was used as the secondary antibody. Antibody binding was visualized by a standard streptavidin immunoperoxidase reaction. Immuno-reactivity in the cytoplasma and cytoplasmic membrane of PTP4A3 was evaluated.

Evaluation immunoreactivity was carried out independently by three experienced pathologists without any knowledge of the clinical data. The IHC score was assessed according to the percentage of positive cells and the intensity of cytoplasmic reactivity. These averaged immunoreactivity values were stratified into five scoring groups: -, not detected; ±, <10% positive cells; +, 10–20% weakly to moderately positive cells; ++, 10–20%intensely positive cells or 20–50% weakly positive cells; and +++, 20–50% positive cells with moderate to marked reactivity or >50% positive cells. In the statistical analysis, ± were considered negative, + and above were considered positive.

### Quantification of CD3+ and CD8+TILs

Each entire section was evaluated for CD3+ and CD8+ TILs by two pathologists blind to PTP4A3 expression. The intratumor and invasive margins were identified by the immunohistochemistry. Each positive cell was manually counted under 40 ×magnification field in 5 independent tumor fields or invasive margins with most abundant TILs. The consecutive sections were stained for CD3 and CD8.

### TIMER Database Analysis

Tumor Immune Estimation Resource (TIMER, https://cistrome.shinyapps.io/timer/) was developed to evaluate the abundance of immune infiltrates using 10,897 cancer samples from TCGA (31, 32). TIMER gene modules were utilized to investigate PTP4A3 expression in diverse tumors and the relationship between PTP4A3 expression and the abundance of immune infiltrates. In addition, correlation modules were used to evaluate the relationship of PTP4A3 expression with gene biomarkers of tumor-infiltrating immune cells. These gene biomarkers have been previously studied (35–37).

### GEPIA Database Analysis

We confirmed the correlation of PTP4A3 expression and tumor infiltration of immune cells with the Gene Expression Profiling Interactive Analysis (GEPIA, http://gepia.cancer-pku.cn/index.html) (38). We also assessed the prognostic value, including OS and DFS, of gene expression in diverse tumors with this database. Meanwhile, gene expression correlation was investigated based on TCGA expression data. Correlation analyses were conducted in both cancer and paired normal tissues. The correlation coefficient was calculated by the Spearman method. PTP4A3 was represented on the X-axis, and related biomarker genes were utilized for the Y-axis.

### Statistical Analysis

Results from the Oncomine database included fold change, P-value, and gene rank. The PrognoScan and GEPIA databases produced HR and P-values or Cox P-values according to a log-rank test. The correlation between PTP4A3 expression and other related immune genes was investigated using the Spearman method and the correlation strength was categorized according to r values. R values of 0.80-1.00 were deemed very strong, r values of 0.60-0.79 were strong, r values of 0.40-0.59 were moderate, r values of 0.20-0.39 were weak, and r values of 0.00-0.19 were categorized as very weak. P-values less than 0.05 were considered statistically significant. Chi-square test and nonparametric test were utilized to analyze the Categorical data by SPSS 19.0 software (SPSS Inc., Chicago, IL, USA).

## Results

### PTP4A3 mRNA Expression Level in Diverse Tumors and Adjacent Normal Tissues

**Figure 1:**
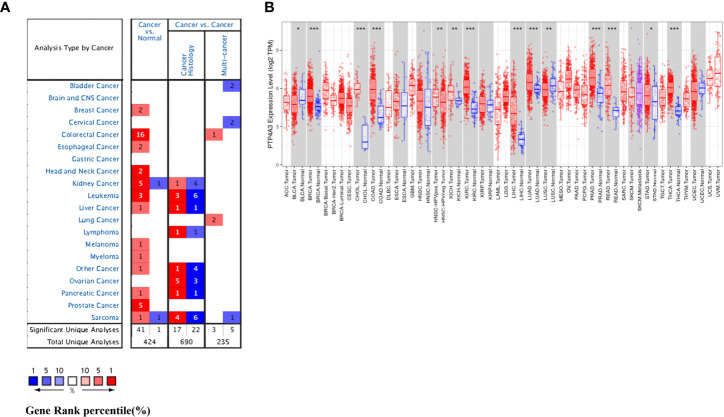
PTP4A3 mRNA levels in various tumor tissues and adjacent normal tissues. **(A)** Differential PTP4A3 mRNA levels between tumor and adjacent normal tissues across various cancer types in the Oncomine database (threshold P-value < 0.001, fold change 1.5, gene rank top 5%). **(B)** Differential PTP4A3 expression levels between tumor and adjacent normal tissues in the TIMER datasets. Blue code represents low expression of indicated tumor. Red code represents high expression of indicated tumor (*P < 0.05, **P < 0.01, ***P < 0.001).

We investigated differential PTP4A3 mRNA levels in tumor and normal tissues across various cancer types in the Oncomine database. Compared with adjacent normal tissues, PTP4A3 mRNA levels were elevated in most cancer types, including breast, colorectal, esophageal, head and neck, kidney, leukemia, liver, melanoma, myeloma, pancreatic, prostate, and sarcoma. However, PTP4A3 mRNA levels were lower in kidney and sarcoma cancer in other datasets (**Figure 1A**). PTP4A3 mRNA levels in diverse tumors are detailed in **Supplementary Table 1**.

PTP4A3 expression in diverse tumor types was further assessed using the TCGA and TIMER databases. Differential PTP4A3 expression in tumors compared with adjacent normal tissues in the TCGA datasets is presented in **Figure 1B**. Consistent with the results from the Oncomine database, PTP4A3 expression was significantly elevated in breast invasive carcinoma (BRCA) cholangiocarcinoma (CHOL), colon adenocarcinoma (COAD), kidney renal clear cell carcinoma (KIRC), liver hepatocellular carcinoma (LIHC), lung adenocarcinoma (LUAD), prostate adenocarcinoma (PRAD) rectum adenocarcinoma (READ) and thyroid carcinoma (THCA) relative to adjacent normal tissues (*P*<0.001). PTP4A3 expression was also slightly elevated in chRCC and stomach adenocarcinoma (STAD) compared with adjacent normal tissues (*P*<0.01). However, PTP4A3 expression was significantly lower in urothelial carcinoma (BLCA), and squamous cell lung carcinoma (LUSC) compared with normal tissues. Notably, PTP4A3 expression was significantly higher in head and neck squamous cell carcinoma (HNSC) HPV-positive cancer tissues compared with HNSC HPV-negative cancer tissues (**Figure 1B**). Single cell RNA sequencing has shown that HNSC HPV-positive and HNSC HPV-negative patients have distinct immune gene signatures, indicating that PTP4A3 may be correlated with immune-related genes (39, 40).

### Prognostic Value of PTP4A3 in Multiple Cancer Types

**Figure 2:**
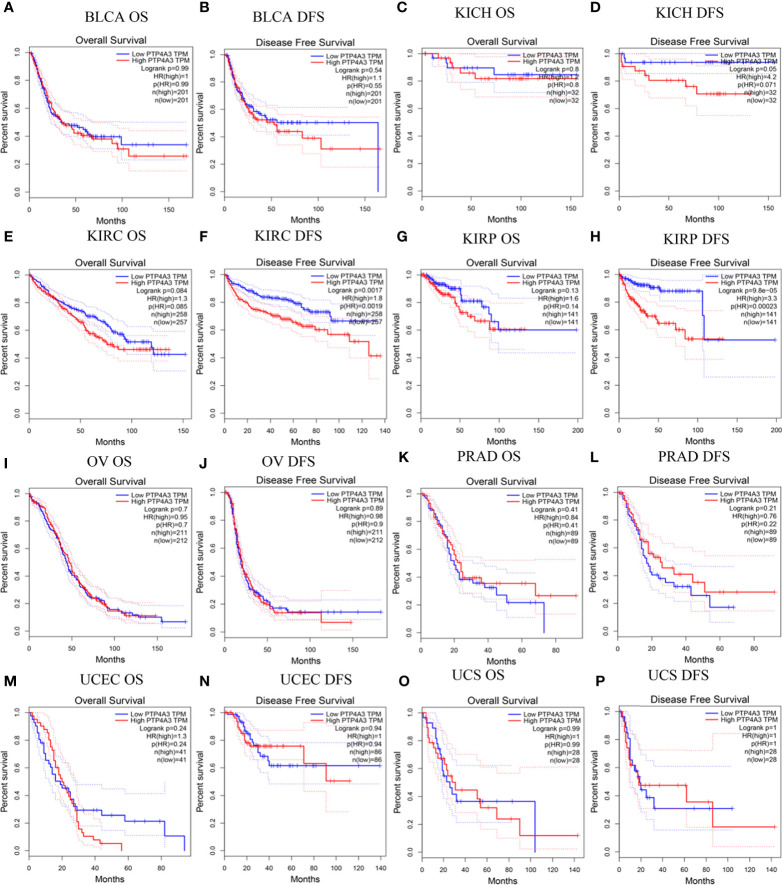
Correlation of PTP4A3 expression level with prognosis values in urothelial tract cancer in the GEPIA database. OS and DFS in patients with differential PTP4A3 expression in bladder urothelial carcinoma (BLCA) **(A, B)**, chRCC (KICH) **(C, D)**, ccRCC(KIRC) **(E, F)**, pRCC (KIRP) **(G, H)**, ovarian serous cystadenocarcinoma (OV) **(I, J)**, prostate adenocarcinoma (PRAD) **(K, L)**, uterine corpus endometrial carcinoma (UCEC) **(M, N)**, uterine carcinosarcoma (UCS) **(O, P)**. OS, overall survival; DFS, disease free survival.

To better understand the impact of PTP4A3 expression on clinical outcomes, the association between PTP4A3 expression and prognosis in 33 cancer types was analyzed using the GEPIA database. OS and DFS in urothelial cancer are presented in **Figure 2**. PTP4A3 expression and OS were not correlated in BLCA, chRCC, KIRC, pRCC (KIRP), ovarian serous cystadenocarcinoma (OV), PRAD, uterine corpus endometrial carcinoma (UCEC) and uterine carcinosarcoma (UCS). High PTP4A3 expression was correlated with unfavorable DFS in KIRC and KIRP (HR=1.8, *P*=0.0017; HR=3.3, *P*=9.8E-05, respectively). The correlations of PTP4A3 expression and OS and DFS in 24 other types of tumors are shown in **Supplementary Figure 1**. High PTP4A3 expression was also correlated with decreased OS in acute myeloid leukemia (AML), lower grade glioma (LGG), LUSC and STAD. In addition, high PTP4A3 expression was correlated with decreased OS and DFS in uveal melanoma (UVM). Interestingly, LUAD was the only tumor type in which low PTP4A3 expression was correlated with decreased DFS.

Survival outcomes were further analyzed using the PrognoScan database. Correlations between PTP4A3 expression and clinical outcomes from 12 cancer types in 154 databases are displayed in **Supplementary Table 2**. Significant survival outcomes (Cox *P*<0.05) are shown in **Supplementary Figure 2**. PTP4A3 expression and survival outcomes were significantly correlated in AML(GSE12417-GPL96), BLCA (GSE5287 and GSE13507), glioblastoma (GSE7696), breast cancer (GSE9893, GSE9195 and GSE9195), eye cancer (GSE22138), MGH-glioma, non-small cell lung cancer (NSCLC) (GSE4716-GPL3696), ovarian cancer (GSE9891) and skin cancer (GSE19234) (**Supplementary Figures 2A–L**). These results indicated that high PTP4A3 expression is significantly associated with poorer clinical outcomes in many tumors types, including KIRC and KIRP.

### PTP4A3 Plays as an Oncogene and Regulates the Cytokines in RCC

**Figure 3:**
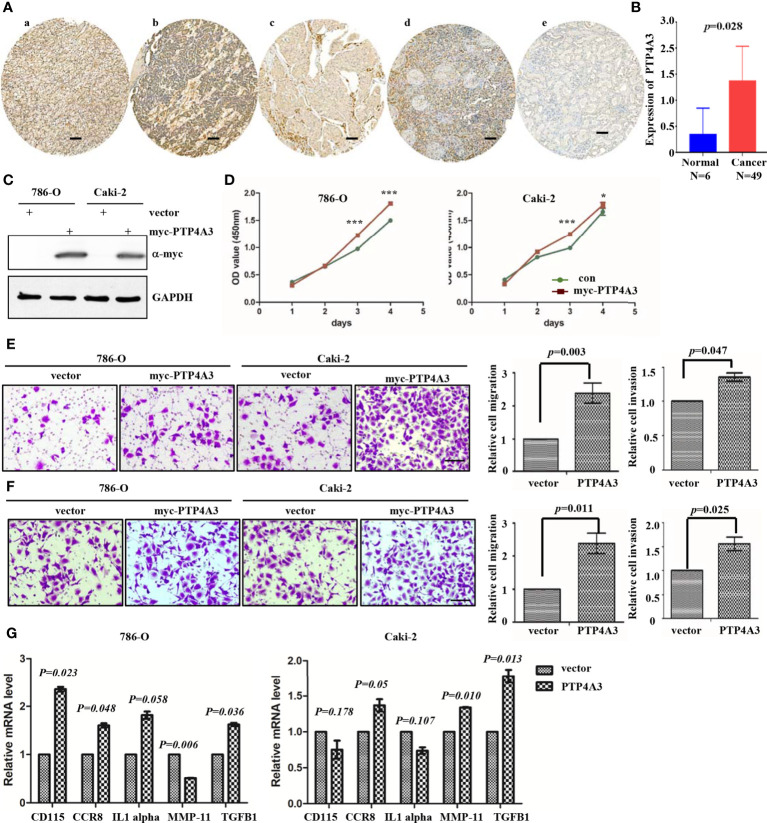
Immunohistochemical staining of PTP4A3 in kidney cancer tissues than the normal tissues. **(A)** Representative IHC stain in kidney tumor tissue array. PTP4A3 expression in KIRC (a), KIRP (b), KICH (c), kidney squamous cancer (d) and kidney normal tissues (e). The Scale bar is 50μM. **(B)** Differential expression of PTP4A3 in kidney cancer and normal tissues. *P*=0.028. **(C)** Ectopic expression of myc-PTP4A3 in 786-O and Caki-2 cell line. GAPDH served as loading control. **(D)** Cell proliferation in 786-O and Caki-2 cells with myc-PTP4A3 and vector expression. All data are mean ± SE of three independent experiments. *P < 0.05, ***P < 0.001. **(E, F)** Representative cell migration and invasion in 786-O and Caki-2 with myc-PTP4A3 and control. All data are mean ± SE of three independent experiments. *P < 0.05, ***P < 0.001. **(G)** The mRNA level of cytokines, CD115, CCR8, IL1 alpha, MMP-11 and TGFB1 in PTP4A3 expressing 786-O and Caki-2 cells. The values are the mean and standard deviation. *P < 0.05, n=3.

To further explore the mechanism of PTP4A3 in RCC, we stained the PTP4A3 in the kidney cancer tissue array which containing 5 different tumor histological subtypes and 6 kidney normal tissues. As shown in **Figures 3A, B**, PTP4A3 expression is obviously higher in the kidney tumor tissues (n=49) compare with the normal tissues (n=6) (*P*=0.028). The representative high expression of PTP4A3 in KIRC, KIRP and KICH, kidney squamous cancer and kidney normal tissues (**Figures 3Aa–e**). And there was no significantly difference in the various histological subtype, such as KIRC, KIRP and other types. (*P*=0.538, **Table 1**). We also calculated the association of PTP4A3 in general clinicopathological features, there was no significant difference found in gender, age, Fuhrman grade, TNM stage and lymph node metastasis (**Table 1**). Notably, PTP4A3 expression was slightly higher in the Fuhrman grade 3 compare with the expression in Grade 1 and 2(77.8% VS 40%, P=0.139). Although there was not rigorous statistic significant, PTP4A3 expression probably indicated the high grade, high recurrence and poor survival.

**Table 1:** PTP4A3 expression and clinical clinicopathological features in the renal cell carcinoma.

Variable		PTP4A3 Expression		*P* Value
		Negative (N = 26)	Positive (N = 23)	
Gender				
	Female	6	6	0.807
	Male	20	17	
Age,year				
	<60	19	14	0.363
	≥60	7	9	
				
Tumor histology				
	KIRC	13	14	0.538
	KIRP	5	2	
	Others	7	7	
				
Fuhrman grade				
	1	7	7	0.139
	2	8	3	
	3	2	7	
	missing	9	6	
TNM stage				
	I	7	6	0.821
	II	14	14	
	III	5	3	
lymph node metastasis				
	No	24	22	0.626
	Yes	2	1	

Results from the GEPIA and Prognoscan database also shown that PTP4A3 may play as an oncogene in the RCC. We utilized the lentivirus infection to ectopic expression of PTP4A3 in 786-O and Caki-2 cell lines. Ectopic expression of myc-PTP4A3 in 786-O and Caki-2 were detected by Myc-tagged antibody (**Figure 3E**). Overexpression of myc-PTP4A3 promoted proliferation of the 786-O and Caki-2 cell lines at 72, 96 hours in CCK-8 experiments (**Figure 3D**). Overexpression of PTP4A3 also triggered the metastasis phenotype of 786-O or Caki-2 cell lines (**Figures 3E, F**). Stable expression of PTP4A3 elevated the secretion level and mRNA level of IL-1alpha in colon cancer cell lines. And IL-1RN (IL-1 alpha inhibitor) decreased the migration and invasion of PTP4A3-stable expression cells (22). Therefore, we detected the cytokines (CD115, CCR8, IL-1 alpha, MMP-11 and TGFB1) in 786-O and Caki-2 cell lines with PTP4A3 stable expression. As shown in **Figure 3G**, ectopic expression of PTP4A3 increase the mRNA level of TGFB1 and CCR8 in 786-O and Caki-2 cells (p<0.05). But ectopic expression of PTP4A3 only elevated the CD115 (*P*=0.023) and IL-1 alpha (*P*=0.058) in 786-O cells, and slightly downregulated the CD115 (*P*=0.178) and IL-1 alpha (*P*=0.107) of Caki-2 cells. Expression of PTP4A3 downregulated the mRNA of MMP-11 in 786-O cells (*P*=0.006), and upregulated MMP-11 in Caki-2 cells (*P*=0.010). In conclusion, high expression of PTP4A3 not only promotes the cell proliferation, migration and invasion, but also increases the mRNA of cytokines TGFB1 ;and CCR8 in RCC cell lines. These results indicated the multiple roles of PTP4A3 in renal cancers, it extended the oncogene role of PTP4A3 to the regulation of cytokines and immune microenvironments.

### PTP4A3 Expression Is Associated With CD3+ and CD8+ Intratumor T Cells in Renal Cancer

**Figure 4:**
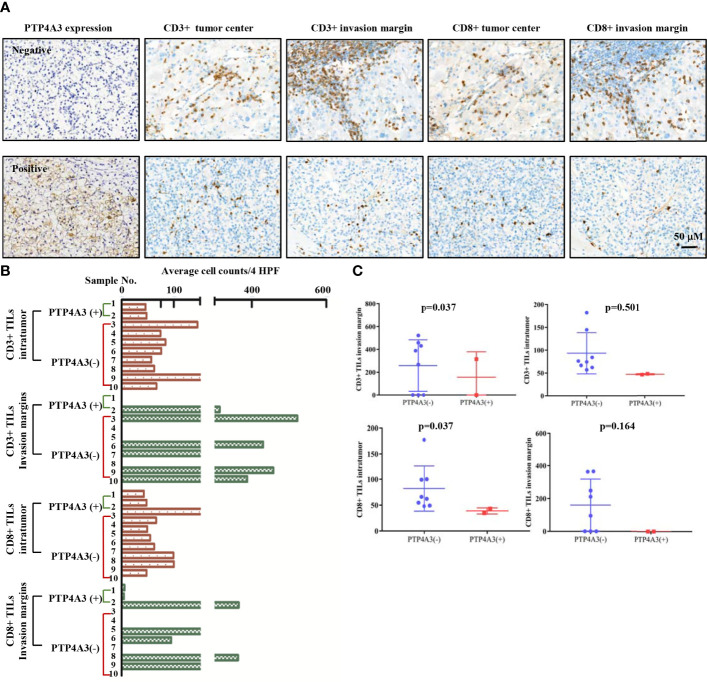
PTP4A3 expression associates with low CD3+/CD8+ infiltration in renal cancers. **(A)** Representative IHC stain in 10 renal tumor tissues. PTP4A3 negative stains (upper panel) and positive stains (lower panel) with CD3+ intratumor stains, CD3+ invasion margins stains, CD8+ intratumor stains, CD8+ invasion margins stains, 20× magnification, scale bar, 50μM. **(B)** CD3+/CD8+ infiltration in tumor center (intratumor) and invasion margins in the PTP4A3 negative and positive tissues. All data were manually counted of positive cells in 4 HPF in each tumor field. **(C)** Histogram of the CD3+ and CD8+ intratumor and invasion margins infiltrations in renal cancers. All data are mean ± SE.

TGFB1 expressing and FOXP3 expressing on Treg cell is important in the maintenance of immune homeostasis. Antitumor effect of PTP4A3-target monoclonal antibody was described in gastric cancer (27). Immunotherapy combined with target therapy began the new era of malignant tumor such as lung cancer, gastric cancer (41–43). Considering tissue array is not suitable for the research of immune cell infiltration, we stained the CD3 (general T cell markers) and CD8+ T cells in KIRC surgery sections (n=10) to further explore the role of PTP4A3 in immnuo-target combined therapy. The representative IHC stains were shown in **Figure 4A**. Each count of CD3+/CD8+ intratumor and CD3/CD8+ invasive margins were shown in **Figure 4B**, divided by the PTP4A3 expression in the individual section. Compared with 8 negative PTP4A3 expression cases, the 2 positive PTP4A3 expression cases had lower CD3+ TILs intratumor counts (75.2(57.0,182)/4 HPF VS 47.2(46.3,48.0)/4 HPF, *P*=0.037) and CD8+ TILs intratumor counts (64.2(48.0,177.3)/4 HPF VS 38.9(34.7,43.0)/4 HPF, *P*=0.037). There were not significant differences in the counts of CD3+ (327.5(0,522.0)/4 HPF VS 157.0(0,314.0)/4 HPF, *P*=0.501) and CD8+ (153.0(0,365.0)/4 HPF VS 0(0, 0)/4 HPF, *P*=0.164) of invasion margins in the PTP4A3 negative and positive groups (**Figure 4C** and **Table 2**). Therefore, PTP4A3 associated with low CD3+/CD8+TILs and indicated poor prognosis in in renal cancers.

**Table 2:** Descriptive statistics for numbers of CD3+ TILs and CD8+ TILs in patients with KIRC.

	PTP4A3(-)			PTP4A3(+)		
	Mean	Median	Range	Mean	Median	Range
CD3+ TILs intratumor	93.38	75.15	57-182	47.15	47.15	46.3-48
CD3+ TILs invasion margin	258.25	327.50	0-522	157.00	157.00	0-314
CD8+ TILs intratumor	82.08	64.15	48-177.3	38.85	38.85	34.7-43.0
CD8+ TILs invasion margin	160.25	153.00	0-365	0.00	0.00	0.00

### PTP4A3 Expression Is Associated With Immune Infiltration in Renal Cancer

Preliminary experiments in **Figure 4** showed PTP4A3 may associate with T cell infiltration in renal cancer. Renal cancer samples were not easy to collect, especially the relative low incidence subtype. Therefore, we utilized the TIMER database to comprehensive explore the relation of PTP4A3 and immune infiltration. Previous research has revealed that tumor-infiltrating lymphocytes could serve as an independent prognostic predictor in various cancers (44, 45). Using the databases (PrognoScan and GEPIA) and cell model, oncogene PTP4A3 expression was found to be correlated with decreased survival in KIRC and KIRP and promoted cell proliferation, migration and invasion (**Figures 2** and **3**).

**Figure 5:**
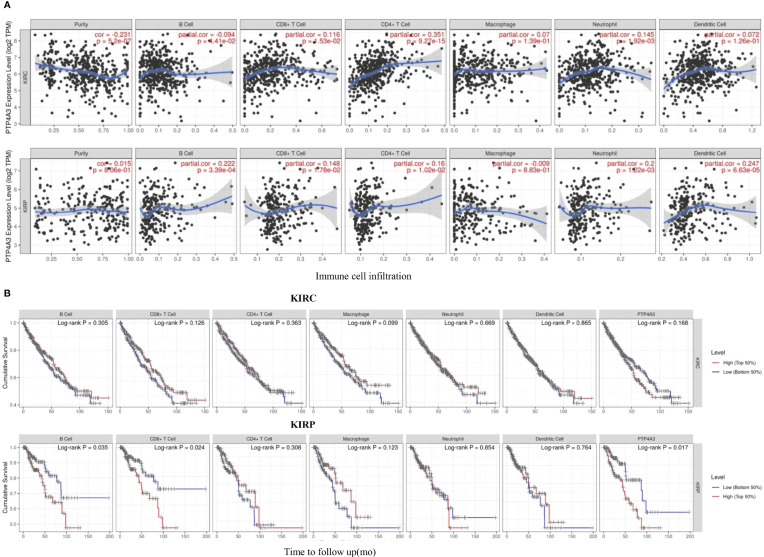
PTP4A3 expression level is correlated with immune cell infiltration in KIRC and KIRP. **(A)** PTP4A3 expression is correlated with immune cell infiltration in KIRC and KIRP. **(B)** Kaplan-Meier plots of immune cell infiltration and PTP4A3 expression levels in KIRC and KIRP.

Consistent with the unique immune gene profiles of renal cancer subtypes, the correlation of PTP4A3 expression with immune cell infiltration was distinct in KIRP and KIRC. In KIRC, significant correlations were observed between PTP4A3 expression and tumor purity (R=-0.231, *P*=5.2E-07), B cells (R=-0.094, *P*=4.41E-02), CD8+ T cells (R=0.116, *P*=1.53E-02), CD4+ T cells (R=0.351, *P*=9.27E-15) and neutrophils (R=0.145, *P*=1.92E-03), but not with macrophages (R=0.007, *P*=1.39E-01) or dendritic cells (R=0.072, *P*=1.26E-01). Similarly, in KIRP, PTP4A3 expression was correlated with B cells (R=0.222, *P*=3.39E-04), CD8+ T cells (R=0.148, *P*=1.78E-02), CD4+ T cells (R=0.16, *P*=1.02E-02), neutrophils (R=0.200, *P*=1.22E-03) and dendritic cells (R=0.247, *P*=6.63E-05), but not with tumor purity (R=0.015, *P*=8.06E-01) or macrophages (R=-0.009, *P*=8.83E-01) (**Figure 5A**). These results were partially accord with the CD3/CD8 staining results in real world.

Kaplan-Meier curves showing immune cell infiltration and PTP4A3 expression in KIRC and KIRP were generated using the TIMER database. B cell infiltration (*P*=0.035), CD8+T cell infiltration (*P*=0.024), and PTP4A3 expression (*P*=0.017) were significantly associated with survival in KIRP (**Figure 5B**). However, there was no significant relationship between immune cell infiltration (including B cells, CD8+T cells, CD4+ T cells, neutrophils, macrophages, and dendritic cells) and survival in KIRC. Taken together, these results suggest that PTP4A3 is involved with regulating immune cell infiltration in KIRP and KIRC and, along with B cell infiltration and CD8+ T cell infiltration, influences clinical outcomes in KIRP.

### Correlation Between PTP4A3 Expression and Immune Marker Sets

To further evaluate the association between PTP4A3 expression and immune cell infiltration, the TIMER and GEPIA databases were utilized to investigate the relationship between PTP4A3 and immune marker sets in KIRP and KIRC.

**Figure 6:**
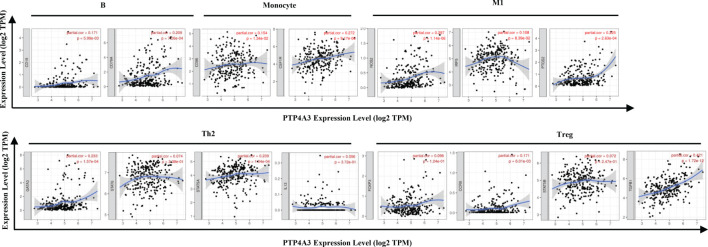
Correlation of PTP4A3 expression levels with gene markers of immune cells in KIRP from the TIMER database. Scatterplots of relationships between PTP4A3 expression levels and gene markers of B, monocytes, M1, Th2 and Treg cells in KIRP.

The association between PTP4A3 expression and immune marker sets of B cells, monocytes, TAMs, M1 macrophages, M2 macrophages, neutrophils, NK cells, dendritic cells, general T cells and CD8+ T cells were examined, both in KIRP and KIRC. Various T cells, including Th1 cells, Th2 cells, Tfh cells, Th17 cells, Tregs and exhausted T cells were also examined (**Supplementary Table 3**). The screen standard was defined as Cor>0.2, *P*<0.05 to identify PTP4A3-associated immune cell markers with or without tumor purity adjustment in KIRP and KIRC. Without tumor purity adjustment, PTP4A3 expression was correlated with CD115 in monocyte markers (Cor=0.215, *P*=2.38E-04), inducible nitric oxide synthase (INOS) in M1 macrophage markers (Cor=0.265, *P*=4.90E-06), STAT5A (Cor=0.231, *P*=7.26E-05) in Th2 markers and TGFB1 (Cor=0.421, *P*=1.72E-12) in Treg markers in KIRP (**Supplementary Table 3**). Distinct from what was observed in KIRP, PTP4A3 was associated with many immune gene markers in KIRC, including Cox2 in M1 macrophage markers (Cor=0.242, *P*=1.56E-08), CCR7 in neutrophil markers (Cor=0.271, *P*=2.10E-10), BDCA-1 (Cor=0.208, *P*=1.20E-06) and BDCA-4 (Cor=0.384, *P*=3.42E-20) in dendritic cell markers, T-bet (Cor=0.279, *P*=5.60E-11) and STAT4 (Cor=0.229, *P*=9.00E-08) in Th1 markers, BCL6 (Cor=0.249, *P*=5.55E-09) in Tfh markers, STAT3 (Cor=0.249, *P*=5.84E-09) in Th17 markers, TGFB1 (Cor=0.510, *P*=1.37E-36) in Treg markers and GZMB (Cor=0.215, *P*=5.46E-07) in T cell exhaustion markers (**Supplementary Table 3**). After adjusting for tumor purity, in both KIRP and KIRC, PTP4A3 expression was associated with M1 macrophage markers (INOS, Cor=0.297, *P*=1.14E-06, Cor=0.300, *P*=4.83E-11; COX2, Cor=0.225, *P*=2.63E-04, Cor=0.233, *P*=4.03E-07), Th17 markers (STAT3 Cor=0.225, *P*=2.74E-04, Cor=0.221, *P*=1.62E-06) and Treg markers (TGFβ Cor=0.378, *P*=3.75E-11, Cor=0.502, *P*=8.27E-31). CD79A in B cell markers (Cor=0.209, *P*=7.26E-04), CSF1R in monocyte markers (Cor=0.272, *P*=9.17E-06), VSIG4 in M2 macrophage markers (Cor=0.201, *P*=1.17E-03), CD11b in neutrophil markers(Cor=0.203, *P*=1.03E-03), HLA-DPA1 (Cor=0.210, *P*=6.99E-04) and BDCA-1 (Cor=0.225, *P*=2.65E-04)in dendritic cell markers, GATA3 (Cor=0.233, *P*=1.57E-04) and STAT5A (Cor=0.239, *P*=1.04E-04) in Th2 markersand LAG3 (Cor=0.216, *P*=4.85E-04) and GZMB (Cor=0.221, *P*=3.58E-04) in T cell exhaustion markers were correlated with PTP4A3 expression in KIRP (**Supplementary Table 3** and **Figure 6**). Weak correlations were observed between PTP4A3 expression and B cell markers (CD19), monocyte markers (CD68), NK cell markers (KIR2DL4, KIR3DL1, KIR3DL2 and KIR2DS4), dendritic cell markers (HLA-DPB1 and HLA-DRA), Th1 markers (TNF), Th2 markers (GATA3, STAT5A), Treg markers (CCR8), and exhausted T cell markers (PD1) in KIRP (*P*<0.01; **Supplementary Table 3**). TBX21 in Th1 markers was strongly correlated with PTP4A3 expression in KIRC (Cor=0.234, *P*=3.75E-07). PTP4A3 was weakly correlated with neutrophils (CCR7), NK cell markers (KIR2DL1, KIR3DL1, KIR3DL2 and KIR2DS4), dendritic cell markers (HLA-DRA and BDCA-1), Th1 (STAT4 and IFN-γ), Th2 (STAT6 and IL13), Treg (STAT5B) and T cell exhaustion (TIM-3 and GZMB) in KIRC. The correlation of PTP4A3 expression and B cell, monocyte, M1 macrophage, Th2 and Treg markers were confirmed using the GEPIA database, which compiled data from KIRP tumors and normal tissues (**Table 3**). No correlations were observed between PTP4A3 expression and M2 macrophage, neutrophil, or T cell exhaustion markers in the GEPIA database, but B cells, monocytes and Th2 markers were correlated with PTP4A3 expression in KIRP. Together, these findings confirmed the relationship of PTP4A3 to immune cell infiltration and CD79A, CSF1R, INOS, COX2, STAT5A, FOXP3 and CCR8 in KIRP, suggesting an important immune regulation role of PTP4A3 in the renal cancer microenvironment.

**Table 3:** Correlations between PTP4A3 expression and related gene markers in the GEPIA database.

Description	Gene markers	KIRP			
		Tumor		Normal	
		R	*P*	R	*P*
B cell	CD19	-0.039	5.10E-01	0.130	4.90E-01
	CD79A	0.210	***	0.110	5.50E-01
Monocyte	CD86	0.042	4.80E-01	0.150	4.00E-01
	CD115(CSF1R)	0.190	**	0.110	5.60E-01
M1 Macrophage	INOS(NOS2)	0.250	***	-0.390	*
	IRF5	0.047	4.30E-01	0.190	3.00E-01
	COX2(PTGS2)	0.300	***	-0.093	6.10E-01
Th2	GATA3	0.110	5.30E-02	0.410	*
	STAT6	-0.012	8.50E-01	0.430	*
	STAT5A	0.160	**	0.030	8.70E-01
	IL13	0.014	8.10E-01	0.150	4.10E-01
Treg	FOXP3	0.190	**	0.330	6.30E-02
	CCR8	0.190	**	0.220	2.20E-01
	STAT5B	-0.034	5.70E-01	-0.100	5.80E-01
	TGFβ(TGFB1)	0.370	***	0.400	*

*P < 0.05, **P < 0.01, ***P < 0.001.

KIRP, papillary renal cell cancer.

## Discussion

PTP4A3 is a 22kD tyrosine phosphatase and is highly homologous with PTP4A1 and PTP4A2. While PTP4A1 is expressed ubiquitously, PTP4A2 and PTP4A3 are specifically expressed in heart and skeletal muscles. PTP4A3 is a dual phosphatase and is known to act on ezrin, cytokeratin and integrin β1 (46–48). PTP4A3 is involved in the regulation of cell proliferation, migration and invasion through the PI3K-AKT-ERK pathway and destabilizes telomeres through the NFкB-RAP1-TRF2 axis (18, 23, 24, 49). Moreover, PTP4A3 contributes to the secretion of inflammatory factors, which recruit more immune cells to the tumor microenvironment. Activation of PTP4A3 expression increases IL-1 alpha secretion through the NF-κB and JAK2-STAT pathway (22). On the other hand, IL-6/8 secreted by TAMs facilitated the metastasis of colon cancer in a PTP4A3-KCNN_4_ dependent manner (30). Research with target monoclonal antibodies and DNA vaccines has indicated that PTP4A3 may collaborate with CTL and Th1 cells in the tumor microenvironment. PTP4A3-zumab recruited B cells, NK cells and macrophages to the PTP4A3+ tumor cells in an FcR-dependent manner (27, 50). Therefore, PTP4A3 could be a potential target for cancer immunotherapy. Clinical trials have shown that immunotherapy in renal cell carcinoma is superior to tyrosine kinase inhibitors, heralding a new era of renal cancer treatment.

As there is no study showing PTP4A3 expression and its potential roles in renal cancer, further experiments were conducted to evaluate whether PTP4A3 play as an oncogene in RCC. In our validation cohort, PTP4A3 expression is significantly elevated in the tumor tissues by the immunohistochemical staining of PTP4A3 in 49 kidney cancers and 6 normal tissues. As for the functional study, ectopic expression of PTP4A3 promotes proliferation, migration and invasion in RCC cell lines. Taken collectively, the above findings firstly suggested that PTP4A3 serve as an oncogene in renal cancer. The metastasis phenotype could be partially inhibited by IL-1alpha inhibitors, and MMP11 and FOXN3 were elevated in the PTP4A3 overexpressing colon cells (22). These immune related cytokines may connect the oncogene PTP4A3 with the immune infiltration. We also tried to verify the correlation of PTP4A3 and IL-1 alpha in RCC cells, but the mRNA level of IL-1 alpha showed different changes in 786-O and Caki-2 cell. Considering the possible inconsistent of mRNA level and secretion level in supernatant, the cytokines in supernatant should be detected by the ELISA to further confirm the correlations. Luckily, we found that PTP4A3 upregulated the mRNA of TGFB1 in both 786-O and Caki-2 cells. TGFB1 expressed on the Treg cell surface and maintain the immune hemostasis. The correlation of PTP4A3 and TGFB1 were also been confirmed in **Table 3** by the TIMER database (R=0.370, p<0.001). Therefore, it indicated that PTP4A3 may also regulate the immune microenvironment through cytokines TGFB1 in RCC.

In this study, we focused on the association between PTP4A3 and cytotoxic T cells, which have not been jointly reported. CD3+, CD8+ T cells represent T cells, cytotoxic T cells. Our explorations examined the immune infiltrates’ density and the location of immune cell populations in intratumor and invasion margins. In KIRC, we observed negative association between PTP4A3 expression and the density of CD3+ TILs intratumor and CD8+ TILs intratumor in the tumor microenvironment. There was no significant relationship between PTP4A3 expression and the number of CD3+ TILs invasion margins and CD8+ TILs invasion margins. However, we did not assess whether the combination of PTP4A3 and CD3+ and CD8+ T cells have independent prognostic significance in renal cancer. Previous studies reported that high density of CD3+, CD8+ T cells correlated with favorable prognosis in various cancers (51, 52). These findings suggested that immune infiltrates of T cells play a protective role. The role of CD8+ T cells in renal cancer is controversial, maybe attribute to technical factors such as antibodies used to examine CD8+ T cells and different tumor location detected (intratumor vs invasion margins TILs) (52, 53).

In general, the tumor-infiltrating T cells play vital roles in renal cancer, including cytotoxic T cells, memory T cells, regulatory T cells, and so on (54, 55). In the future work, the relationship between PTP4A3 expression and B cells and other T cells including CD4+ T cells, CD45RO+ memory T cells, regulatory T cell need to be explored in renal cancer.

In this study, the role of PTP4A3 in renal cell carcinoma immune microenvironments was explored using online databases, including the Oncomine, TCGA, GEPIA, TIMER and Progscan databases. Consistent with previous findings, PTP4A3 expression was elevated in many tumor types compared with normal tissues, including renal cancer. Kaplan-Meier survival analysis suggested that PTP4A3 expression was correlated with poor DFS but not with OS in KIRC and KIRP. This may be related to the cutoff percentage setting (50% cut-off) and case numbers in the GEPIA database. PTP4A3 expression is higher in gastric cancer than in colon cancer, and the cutoff settings in various cancer types should be adjusted base on the immunohistochemistry of each cancer type. The association of PTP4A3 expression with B cells, CD8+ T cells, CD4+T cells and neutrophil infiltration was confirmed in renal cancer (**Figure 5** and **Supplementary Table 3**) using the GEPIA and TIMER databases. These results were consistent with PRL-3-zumab work from Zeng et al. (50). PTP4A3 is an oncogene related to immune cell infiltration in KIRC and KIRP.

Tumorigenesis and tumor development are complicated processes involving thousands of genes and pathways. Because of this, signal gene signatures are rarely used to predict tumor prognosis. Due to the association of PTP4A3 expression with immune cell infiltration, the relationship of immune markers and PTP4A3 expression was analyzed using the TIMER database to identify gene profile signatures that may be able to predict prognosis and immunotherapy response in KIRP. Correlations were observed between PTP4A3 expression and M1 macrophage immune markers such as INOS and COX2 in both KIRP and KIRC (**Supplementary Table 3** and **Table 3**), suggesting that PTP4A3 may regulate the polarization of M1 macrophages in renal cancer. A positive correlation was observed between PTP4A3 expression and B cell immune marker (CD79A) in KIRP only (**Supplementary Table 3** and **Table 3**), and this result was confirmed with the TIMER database. Dendritic cells can play a vital role in tumor growth and metastasis by inhibiting CD8+ T cell cytotoxicity and promoting Treg cells (56). PTP4A3 expression was correlated with HLA-DRA, BDCA-1, and BDCA-4 in dendritic cells, and with CCR8 and TGFB1 in Treg cells, indicating that PTP4A3 promoted tumor growth and metastasis through the classical PI3K-AKT-PTEN signal pathway and also by regulating the tumor immune microenvironment (18–20). Interestingly, exhausted T cell markers such as PD1, LAG3 and GZMB were also significantly correlated with PTP4A3 expression in KIRP (**Supplementary Table 3**). PD1 is the immune checkpoint receptor expressed on the activated T cells; blocking the PD1/PD-L1 interaction would release immune suppression and trigger immune clearance in tumors. This study is the first reported association of PTP4A3 and PD1 in renal cancer. The combination of PTP4A3 with immune cells is proposed as a predictor for immunotherapy response and prognosis in renal cancers, with potential applications in other cancer types such as NSCLC or gastric cancer as well (57, 58). These findings further confirm the relationship of PTP4A3 expression to immune infiltration in KIRP, suggesting that PTP4A3 influences immune escape in the renal cancer microenvironment. We are confidence in our results base on the various online datasets, therefore we are working on the collection of renal cancer samples and focusing on the immune-related role of PTP4A3 in renal cancer.

In conclusion, PTP4A3 may be a regulator in the tumor immune microenvironment and a useful biomarker for predicting prognosis and immunotherapy response in renal cancer. These results will be verified in our further workings.

## Data Availability Statement

The original contributions presented in the study are included in the article/**Supplementary Material**. Further inquiries can be directed to the corresponding authors.

## Ethics Statement

The studies involving human participants were reviewed and approved by the Ethics Committee of Peking University Cancer Hospital & Institute (Beijing, China). The patients/participants provided their written informed consent to participate in this study. Written informed consent was obtained from the individual(s) for the publication of any potentially identifiable images or data included in this article.

## Author Contributions

QS, ZL, and SL conceived the project and wrote the manuscript. JW and SW managed data acquisition. QF, JW, and SW participated in the data analysis. QS and SL participated in the discussion and language editing. ZL reviewed the manuscript. YZ did the IHC, cell proliferation migration, and invasion experiments during the revised of the manuscript. LZ and QY counted the CD3+ and CD8+ TILs. All authors contributed to the article and approved the submitted version.

## Funding

This research was supported by Natural Science Foundation of China [grant number 81872309], Beijing Hospitals Authority Youth Programme [grant number QML20191109], Zhejiang Provincial Natural Science Foundation of China [grant number LQ21H200001] and General Research Program of Health Department of Zhejiang Province [grant number 2020KY480].

## Conflict of Interest

The authors declare that the research was conducted in the absence of any commercial or financial relationships that could be construed as a potential conflict of interest.

## Publisher’s Note

All claims expressed in this article are solely those of the authors and do not necessarily represent those of their affiliated organizations, or those of the publisher, the editors and the reviewers. Any product that may be evaluated in this article, or claim that may be made by its manufacturer, is not guaranteed or endorsed by the publisher.

## Abbreviations

RCC, renal cell carcinoma; ccRCC or KIRC, clear cell RCC; pRCC or KIRP, papillary cell RCC; chRCC, chromophobe RCC; TCGA, the cancer genome atlas; mRCC, metastatic RCC; PD1, programmed cell death protein 1; PD-L1, programmed death-ligand 1; NK, natural killer; OS, overall survival; T_H_17, T helper; PTP4A3, protein tyrosine phosphatase 4A3; PRL-3, phosphatase of regenerating liver 3; AML, acute myeloid leukemia; TAMs, tumor-associated macrophages; DFS, disease-free survival; TIMER, tumor immune estimation resource; GEPIA, gene expression profiling interactive analysis; BRCA, invasive carcinoma of the breast; CHOL, cholangiocarcinoma; COAD, adenocarcinoma of the colon; LIHC, hepatocellular carcinoma; LUAD, adenocarcinoma of the lung; PRAD, prostate adenocarcinoma; READ, adenocarcinoma of the rectum; THCA, thyroid carcinoma; STAD, gastric adenocarcinoma; BLCA, urothelial carcinoma; LUSC, squamous cell lung carcinoma; HNSC, head and neck squamous cell carcinoma; OV, ovarian serous cystadenocarcinoma; UCEC, uterine corpus endometrial carcinoma; UCS, uterine carcinosarcoma; LAML, acute myeloid leukemia; LGG, low grade glioma; UVM, uveal melanoma; INOS, inducible nitric oxide synthase.
