# Supplementary material for: PTP4A3 Is a Prognostic Biomarker Correlated With Immune Infiltrates in Papillary Renal Cell Carcinoma
Source: Front Immunol. 2021 Sep 23;12:717688. doi: 10.3389/fimmu.2021.717688 (PMC8495008; doi:10.3389/fimmu.2021.717688)
Supplement: Supplementary file 1 [file DataSheet_1.pdf]

Supplementary Figure Legend

Supplementary Figure 1. Relationship of PTP4A3 expression with prognostic values in the remaining types of cancer from GEPIA database. Overall survival and disease free survival curves comparing the high and low expression of PTP4A3 in Adrenocortical carcinoma (ACC) (A-B), Breast invasive carcinoma (BRCA) (C-D), Cervical squamous cell carcinoma and endocervical adenocarcinoma (CESC) (E-F), Cholangio carcinoma(CHOL) (G-H), Colon adenocarcinoma(COAD) (I-J), Lymphoid Neoplasm Diffuse Large B-cell Lymphoma (DLBC) (K-L), Esophageal carcinoma (ESCA) (M-N), Glioblastoma multiforme (GBM) (O-P), Head and Neck squamous cell carcinoma (HNSC) (Q-R), Acute Myeloid Leukemia (AML) (S-T), Lung adenocarcinoma (LUAD) (Y-Z), Lung squamous cell carcinoma (LUSC) (AA-AB), Mesothelioma (MESO) (AC-AD), Pancreatic adenocarcinoma (PAAD) (AE-AF), Pheochromocytoma and Paraganglioma (PCPG) (AG-AH), Rectum adenocarcinoma (READ) (AI-AJ), Sarcoma (SARC) (AK-AL), Skin Cutaneous Melanoma(SKCM) (AM-AN),Stomach adenocarcinoma(STAD) (AO-AP), Testicular Germ Cell Tumors (TGCT) (AQ-AR), Thyroid carcinoma (THCA) (AS-AT), Thymoma (THYM) (AU-AV), Uveal Melanoma (UVM) (AW-AX).

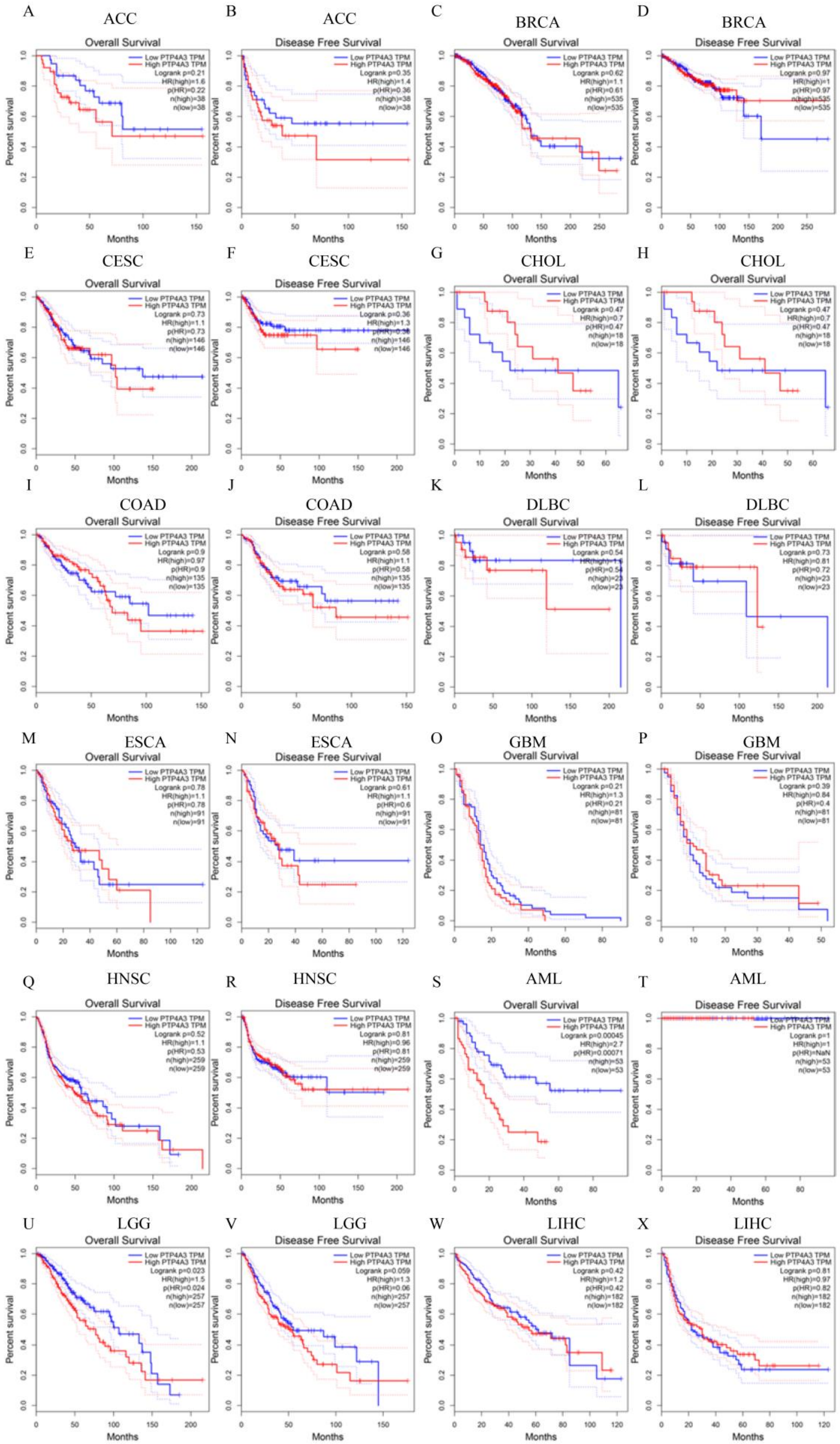

Supplemental Figure 1

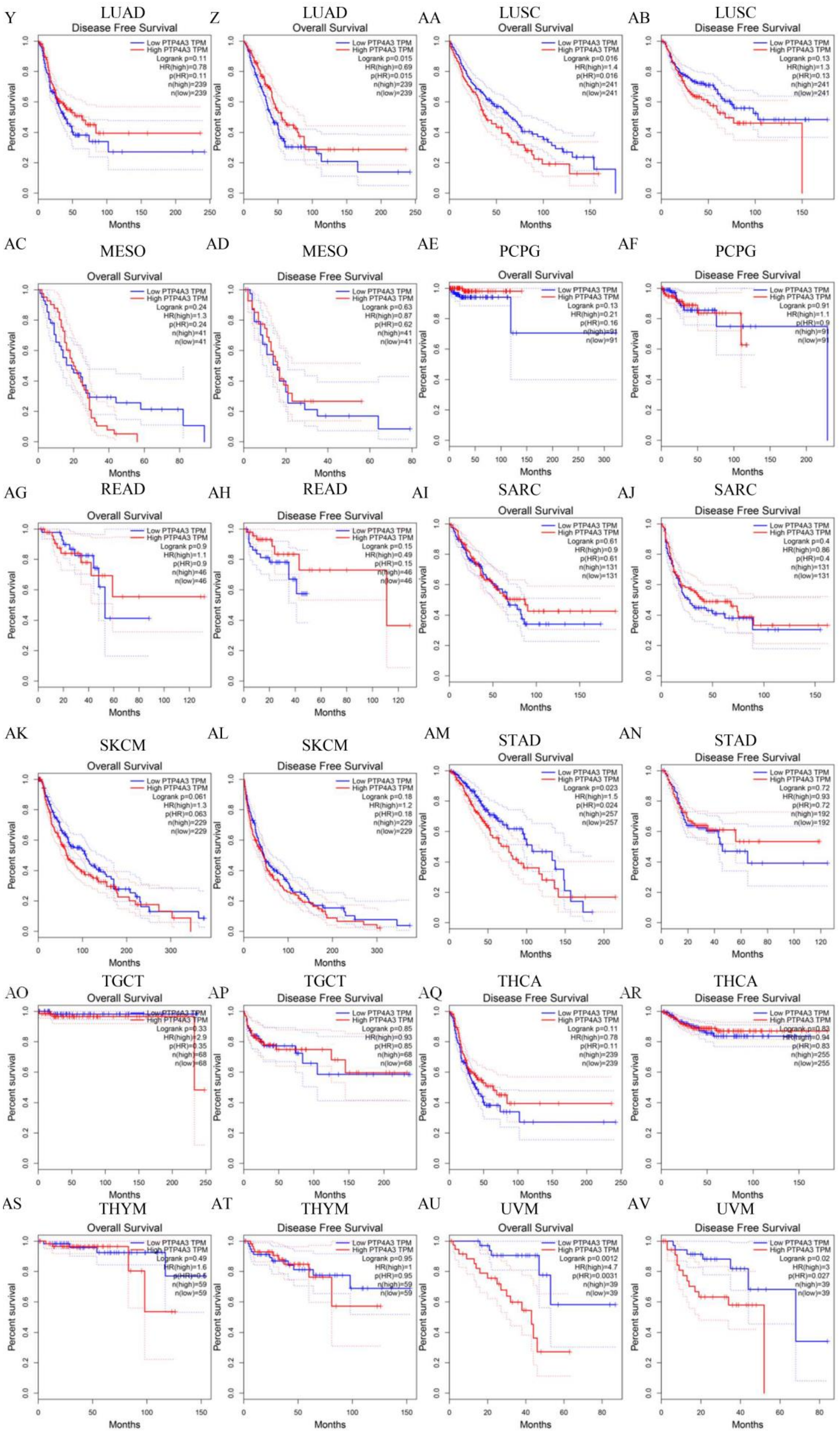

Supplemental Figure 1

Supplementary Figure 2. Correlation of PTP4A3 expression level with prognostic values in various cancer types from the PrognoScan database. Survival curves comparing the high and low expression of PTP4A3 in Acute myeloid leukemia (AML) (A), Bladder Urothelial Carcinoma (BLCA) (B-C), Giloblastoma (D), Breast cancer (E-G), Eye cancer (H), MGH-glioma (I), Non-small cell lung cancer (NSCLC) (J), Ovarian cancer (K), and Skin cancer (L). OS, overall survival; DSS, disease-specific survival; DMFS, distant metastasis-free survival.

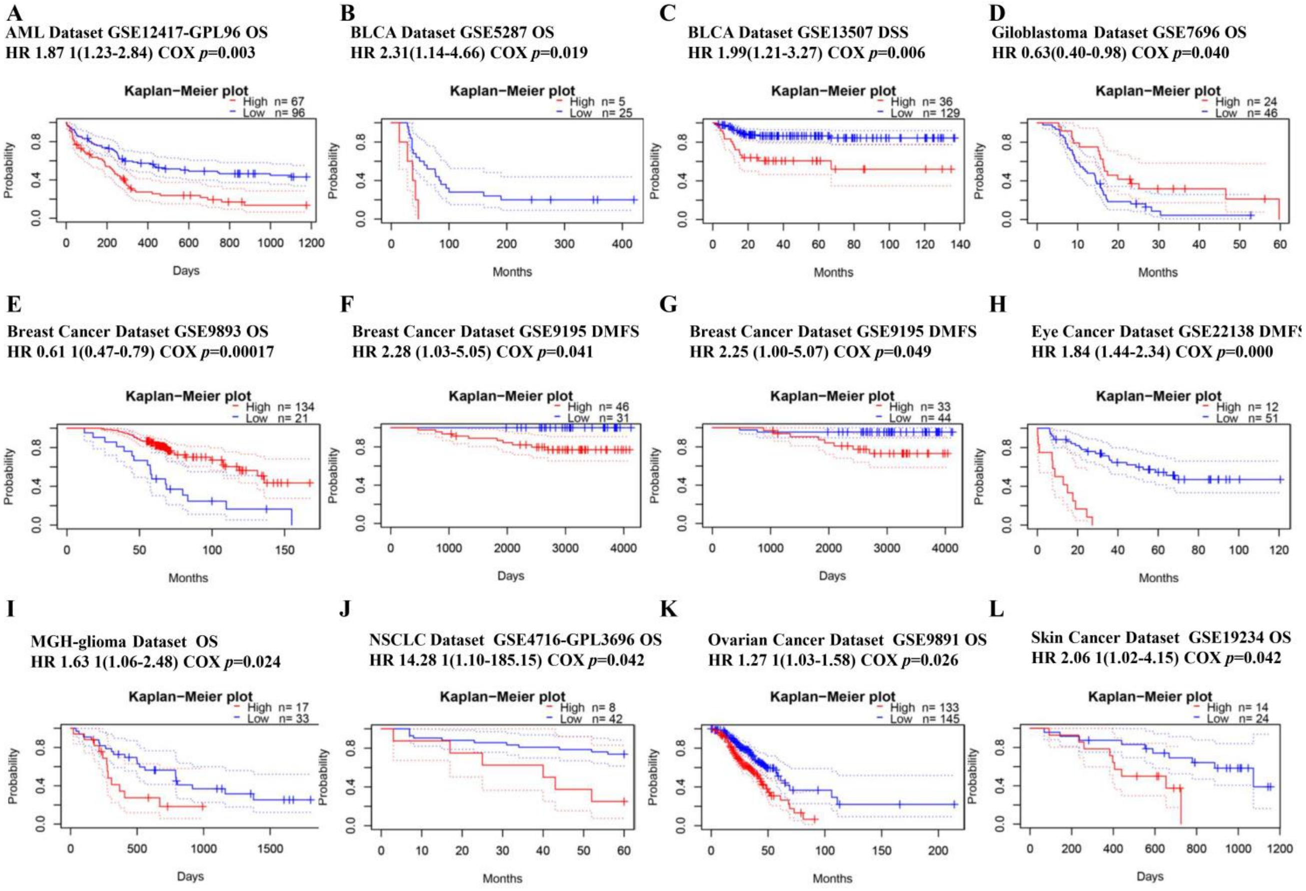

Supplemental Figure 2
