# Supplementary material for: PTP4A3 Is a Prognostic Biomarker Correlated With Immune Infiltrates in Papillary Renal Cell Carcinoma
Source: Front Immunol. 2021 Sep 23;12:717688. doi: 10.3389/fimmu.2021.717688 (PMC8495008; doi:10.3389/fimmu.2021.717688)
Supplement: Supplementary file 2 [file DataSheet_2.docx]

Supplementary Table 1.PTP4A3 expression in cancers verus normal tissue in oncomine database.

| Cancer | Cancer type | P-value | Fold change | Rank (%) | Sample | Reference (PMID) |
| --- | --- | --- | --- | --- | --- | --- |
| Breast | Mucinous Breast Carcinoma | 2.89E-04 | 1.972 | 3% | 65 | TCGA |
|  | Ductal Breast Carcinoma | 2.81E-06 | 2.288 | 4% | 47 | 16473279 |
| Colorectal | Colon Carcinoma | 6.64E-12 | 7.712 | 1% | 15 | 20957034 |
|  | Colon Carcinoma Epithelia | 2.91E-11 | 9.765 | 1% | 15 | 20957034 |
|  | Colon Adenoma Epithelia | 2.61E-07 | 10.291 | 2% | 15 | 20957034 |
|  | Colon Adenoma | 2.29E-06 | 4.932 | 4% | 15 | 20957034 |
|  | Colon Adenoma | 1.34E-18 | 10.632 | 1% | 57 | 18171984 |
|  | Colorectal Carcinoma | 3.62E-13 | 2.732 | 1% | 60 | 20957034 |
|  | Colorectal Adenocarcinoma | 5.74E-13 | 2.786 | 2% | 69 | 20957034 |
|  | Colon Adenocarcinoma | 1.02E-25 | 2.832 | 1% | 123 | TCGA |
|  | Rectal Adenocarcinoma | 2.18E-19 | 2.904 | 3% | 82 | TCGA |
|  | Rectal Adenocarcinoma | 6.55E-32 | 4.472 | 1% | 130 | 20725992 |
|  | Cecum Adenocarcinoma | 3.83E-07 | 2.708 | 3% | 22 | 17615082 |
|  | Colon Mucinous Adenocarcinoma | 2.45E-06 | 2.418 | 4% | 18 | 17615082 |
|  | Rectosigmoid Adenocarcinoma | 1.69E-05 | 2.6 | 4% | 15 | 17615082 |
|  | Rectal Adenocarcinoma | 1.45E-04 | 2.455 | 5% | 13 | 17615082 |
|  | Colon Adenocarcinoma | 2.77E-07 | 1.593 | 5% | 46 | 17615082 |
|  | Colorectal Carcinoma | 7.51E-11 | 3.209 | 4% | 82 | 20143136 |
| Esophageal | Esophageal Squamous Cell Carcinoma | 3.11E-06 | 1.995 | 5% | 34 | 20955586 |
|  | Esophageal Adenocarcinoma | 3.55E-12 | 1.676 | 4% | 103 | 21152079 |
| Head and Neck | Thyroid Gland Papillary Carcinoma | 3.10E-06 | 2.289 | 1% | 18 | 16365291 |
|  | Thyroid Gland Papillary Carcinoma | 3.52E-04 | 1.809 | 2% | 18 | 17296934 |
| Kidney | Renal Oncocytoma | 1.63E-05 | 3.035 | 1% | 9 | 19445733 |
|  | Clear Cell Renal Cell Carcinoma | 4.90E-07 | 2.784 | 2% | 31 | 19445733 |
|  | Chromophobe Renal Cell Carcinoma | 7.94E-04 | 2.645 | 3% | 9 | 19445733 |
|  | Non-Hereditary Clear Cell Renal Cell Carcinoma | 1.58E-09 | 2.433 | 2% | 38 | 19470766 |
|  | Clear Cell Renal Cell Carcinoma | 3.72E-04 | 1.928 | 4% | 18 | 14641932 |
|  | Clear Cell Sarcoma of the Kidney | 6.20E-06 | -5.282 | 2% | 17 | 16299227 |
| Leukemia | B-Cell Acute Lymphoblastic Leukemia | 2.45E-11 | 4.243 | 1% | 27 | 16267031 |
|  | B-Cell Childhood Acute Lymphoblastic Leukemia | 2.18E-66 | 2.609 | 1% | 433 | 20406941 |
|  | B-Cell Acute Lymphoblastic Leukemia | 2.72E-38 | 2.933 | 2% | 221 | 20406941 |
| Liver | Hepatocellular Carcinoma | 1.99E-11 | 2.198 | 4% | 178 | 12058060 |
| Melanoma | Cutaneous Melanoma | 5.98E-04 | 1.953 | 3% | 18 | 18442402 |
| Myeloma | Multiple Myeloma | 3.45E-04 | 3.089 | 4% | 138 | 19396863 |
| Other | Uterine Corpus Leiomyoma | 5.11E-05 | 1.757 | 5% | 77 | 19622772 |
| Pancreatic | Pancreatic Ductal Adenocarcinoma | 2.03E-04 | 1.518 | 3% | 14 | 16103885 |
| Prostate | Prostate Carcinoma | 1.16E-07 | 1.756 | 1% | 26 | 12154061 |
|  | Prostate Carcinoma | 1.18E-04 | 2.665 | 1% | 21 | 19737960 |
|  | Prostate Carcinoma | 8.02E-05 | 1.541 | 3% | 102 | 12086878 |
|  | Prostate Carcinoma | 6.61E-08 | 1.702 | 2% | 87 | 22722839 |
|  | Prostate Adenocarcinoma | 3.47E-05 | 2.071 | 3% | 35 | 12873976 |
| Sarcoma | Gastrointestinal Stromal Tumor | 2.02E-05 | 13.745 | 3% | 25 | 21447720 |
|  | Clear Cell Sarcoma of the Kidney | 6.20E-06 | -5.282 | 2% | 17 | 16299227 |

| Supplementary Table 2. Relation between PTP4A3 expression and patient progonsis of different cancer in Prognoscan database. | | | | | |
| --- | --- | --- | --- | --- | --- |
| Cancer type | Dataset | Endpoint | N | HR [95% CI-low CI-upp] | Cox P |
| Bladder cancer | GSE5287 | OS | 30 | 2.31 [1.14 - 4.66] | 0.020 |
|  | GSE5287 | OS | 30 | 1.34 [0.45 - 4.01] | 0.603 |
|  | GSE13507 | OS | 165 | 1.20 [0.82 - 1.76] | 0.338 |
|  | GSE13507 | DSS | 165 | 1.99 [1.21 - 3.27] | 0.007 |
| Blood cancer | GSE12417-GPL96 | OS | 163 | 1.87 [1.23 - 2.84] | 0.003 |
|  | GSE12417-GPL96 | OS | 163 | 1.63 [1.11 - 2.41] | 0.014 |
|  | GSE12417-GPL570 | OS | 79 | 1.51 [0.94 - 2.43] | 0.087 |
|  | GSE12417-GPL570 | OS | 79 | 1.45 [0.90 - 2.31] | 0.125 |
|  | GSE5122 | OS | 58 | 1.16 [0.86 - 1.56] | 0.334 |
|  | GSE5122 | OS | 58 | 0.91 [0.51 - 1.62] | 0.737 |
|  | GSE8970 | OS | 34 | 0.88 [0.54 - 1.45] | 0.622 |
|  | GSE8970 | OS | 34 | 1.15 [0.55 - 2.41] | 0.714 |
|  | GSE4475 | OS | 158 | 1.20 [0.86 - 1.66] | 0.287 |
|  | GSE4475 | OS | 158 | 1.24 [0.79 - 1.94] | 0.354 |
|  | E-TABM-346 | OS | 53 | 1.42 [0.68 - 2.94] | 0.347 |
|  | E-TABM-346 | EFS | 53 | 1.25 [0.87 - 1.80] | 0.224 |
|  | E-TABM-346 | EFS | 53 | 1.36 [0.69 - 2.67] | 0.369 |
|  | E-TABM-346 | OS | 53 | 1.27 [0.86 - 1.88] | 0.226 |
|  | GSE16131-GPL96 | OS | 180 | 1.02 [0.79 - 1.31] | 0.888 |
|  | GSE16131-GPL96 | OS | 180 | 1.23 [0.73 - 2.08] | 0.437 |
|  | GSE2658 | DSS | 559 | 1.11 [0.97 - 1.28] | 0.131 |
|  | GSE2658 | DSS | 559 | 1.09 [0.99 - 1.19] | 0.085 |
| Brain cancer | GSE4271-GPL96 | OS | 77 | 0.96 [0.74 - 1.23] | 0.736 |
|  | GSE4271-GPL96 | OS | 77 | 0.66 [0.38 - 1.15] | 0.142 |
|  | GSE7696 | OS | 70 | 0.61 [0.39 - 0.98] | 0.039 |
|  | GSE7696 | OS | 70 | 0.63 [0.40 - 0.98] | 0.040 |
|  | MGH-glioma | OS | 50 | 1.63 [1.06 - 2.48] | 0.025 |
|  | GSE4412-GPL96 | OS | 74 | 0.83 [0.57 - 1.21] | 0.324 |
|  | GSE4412-GPL96 | OS | 74 | 0.74 [0.31 - 1.80] | 0.512 |
|  | GSE16581 | OS | 67 | 1.74 [0.23 - 13.10] | 0.589 |
|  | GSE16581 | OS | 67 | 1.37 [0.11 - 17.74] | 0.807 |
| Breast cancer | GSE19615 | DMFS | 115 | 1.43 [0.67 - 3.05] | 0.350 |
|  | GSE19615 | DMFS | 115 | 1.33 [0.48 - 3.70] | 0.585 |
|  | GSE3143 | OS | 158 | 1.38 [0.62 - 3.11] | 0.432 |
|  | GSE7849 | DFS | 76 | 1.00 [0.16 - 6.10] | 0.999 |
|  | GSE12276 | RFS | 204 | 1.19 [0.87 - 1.63] | 0.279 |
|  | GSE12276 | RFS | 204 | 1.24 [0.97 - 1.58] | 0.081 |
|  | GSE6532-GPL570 | DMFS | 87 | 0.73 [0.39 - 1.34] | 0.310 |
|  | GSE6532-GPL570 | DMFS | 87 | 0.66 [0.36 - 1.22] | 0.183 |
|  | GSE6532-GPL570 | RFS | 87 | 0.73 [0.39 - 1.34] | 0.310 |
|  | GSE6532-GPL570 | RFS | 87 | 0.66 [0.36 - 1.22] | 0.183 |
|  | GSE9195 | DMFS | 77 | 2.28 [1.03 - 5.05] | 0.042 |
|  | GSE9195 | DMFS | 77 | 2.25 [1.00 - 5.07] | 0.049 |
|  | GSE9195 | RFS | 77 | 1.98 [0.99 - 3.97] | 0.054 |
|  | GSE9195 | RFS | 77 | 1.94 [0.95 - 3.94] | 0.069 |
|  | GSE12093 | DMFS | 136 | 1.95 [0.85 - 4.47] | 0.115 |
|  | GSE12093 | DMFS | 136 | 1.38 [0.79 - 2.40] | 0.261 |
|  | GSE11121 | DMFS | 200 | 0.72 [0.42 - 1.25] | 0.249 |
|  | GSE11121 | DMFS | 200 | 1.61 [0.72 - 3.59] | 0.242 |
|  | GSE1378 | RFS | 60 | 1.04 [0.54 - 2.00] | 0.914 |
|  | GSE1379 | RFS | 60 | 1.56 [0.87 - 2.80] | 0.132 |
|  | GSE9893 | OS | 155 | 0.61 [0.47 - 0.79] | 0.000 |
|  | GSE2034 | DMFS | 286 | 0.98 [0.81 - 1.18] | 0.802 |
|  | GSE2034 | DMFS | 286 | 1.23 [0.74 - 2.05] | 0.430 |
|  | GSE1456-GPL96 | RFS | 159 | 1.76 [0.98 - 3.17] | 0.058 |
|  | GSE1456-GPL96 | OS | 159 | 0.95 [0.32 - 2.82] | 0.927 |
|  | GSE1456-GPL96 | DSS | 159 | 1.91 [0.95 - 3.83] | 0.068 |
|  | GSE1456-GPL96 | RFS | 159 | 1.28 [0.44 - 3.76] | 0.654 |
|  | GSE1456-GPL96 | OS | 159 | 1.30 [0.74 - 2.30] | 0.361 |
|  | GSE1456-GPL96 | DSS | 159 | 1.52 [0.44 - 5.31] | 0.507 |
|  | GSE7378 | DFS | 54 | 0.29 [0.05 - 1.54] | 0.145 |
|  | GSE7378 | DFS | 54 | 1.09 [0.43 - 2.79] | 0.854 |
|  | E-TABM-158 | RFS | 117 | 0.83 [0.57 - 1.21] | 0.328 |
|  | E-TABM-158 | DSS | 117 | 0.84 [0.54 - 1.31] | 0.434 |
|  | E-TABM-158 | DMFS | 117 | 0.81 [0.51 - 1.30] | 0.386 |
|  | E-TABM-158 | OS | 117 | 0.83 [0.57 - 1.21] | 0.328 |
|  | E-TABM-158 | RFS | 117 | 0.80 [0.53 - 1.23] | 0.319 |
|  | E-TABM-158 | DSS | 117 | 0.82 [0.49 - 1.37] | 0.458 |
|  | E-TABM-158 | OS | 117 | 0.80 [0.53 - 1.23] | 0.319 |
|  | E-TABM-158 | DMFS | 117 | 0.89 [0.52 - 1.51] | 0.662 |
|  | GSE3494-GPL96 | DSS | 236 | 1.00 [0.45 - 2.23] | 0.996 |
|  | GSE3494-GPL96 | DSS | 236 | 1.13 [0.66 - 1.94] | 0.651 |
|  | GSE4922-GPL96 | DFS | 249 | 0.80 [0.51 - 1.23] | 0.308 |
|  | GSE4922-GPL96 | DFS | 249 | 0.72 [0.37 - 1.43] | 0.351 |
|  | GSE2990 | RFS | 125 | 0.80 [0.41 - 1.57] | 0.522 |
|  | GSE2990 | DMFS | 54 | 0.96 [0.63 - 1.46] | 0.854 |
|  | GSE2990 | RFS | 62 | 1.11 [0.63 - 1.95] | 0.708 |
|  | GSE2990 | DMFS | 125 | 1.11 [0.73 - 1.68] | 0.628 |
|  | GSE2990 | RFS | 62 | 0.97 [0.69 - 1.37] | 0.861 |
|  | GSE2990 | RFS | 125 | 0.95 [0.68 - 1.32] | 0.747 |
|  | GSE2990 | DMFS | 54 | 1.13 [0.56 - 2.28] | 0.735 |
|  | GSE2990 | DMFS | 125 | 0.85 [0.37 - 1.96] | 0.708 |
|  | GSE7390 | DMFS | 198 | 1.19 [0.93 - 1.50] | 0.163 |
|  | GSE7390 | RFS | 198 | 1.14 [0.82 - 1.58] | 0.436 |
|  | GSE7390 | OS | 198 | 1.20 [0.94 - 1.54] | 0.151 |
|  | GSE7390 | DMFS | 198 | 1.20 [0.80 - 1.79] | 0.380 |
|  | GSE7390 | OS | 198 | 1.22 [0.80 - 1.86] | 0.350 |
|  | GSE7390 | RFS | 198 | 1.10 [0.91 - 1.33] | 0.334 |
| Colorectal cancer | GSE12945 | OS | 62 | 0.63 [0.28 - 1.44] | 0.275 |
|  | GSE12945 | DFS | 51 | 0.77 [0.06 - 10.04] | 0.843 |
|  | GSE12945 | OS | 62 | 0.81 [0.15 - 4.48] | 0.812 |
|  | GSE12945 | DFS | 51 | 1.31 [0.45 - 3.82] | 0.621 |
|  | GSE17536 | DSS | 177 | 0.90 [0.55 - 1.45] | 0.654 |
|  | GSE17536 | DSS | 177 | 0.78 [0.46 - 1.33] | 0.367 |
|  | GSE17536 | OS | 177 | 0.84 [0.55 - 1.28] | 0.423 |
|  | GSE17536 | OS | 177 | 0.73 [0.46 - 1.16] | 0.179 |
|  | GSE17536 | DFS | 145 | 1.09 [0.61 - 1.96] | 0.776 |
|  | GSE17536 | DFS | 145 | 0.99 [0.52 - 1.88] | 0.966 |
|  | GSE14333 | DFS | 226 | 0.91 [0.60 - 1.38] | 0.662 |
|  | GSE14333 | DFS | 226 | 1.05 [0.81 - 1.38] | 0.697 |
|  | GSE17537 | OS | 55 | 0.51 [0.22 - 1.21] | 0.127 |
|  | GSE17537 | OS | 55 | 0.61 [0.33 - 1.14] | 0.122 |
|  | GSE17537 | DFS | 55 | 0.69 [0.29 - 1.64] | 0.398 |
|  | GSE17537 | DSS | 49 | 0.50 [0.15 - 1.63] | 0.249 |
|  | GSE17537 | DFS | 55 | 0.78 [0.41 - 1.49] | 0.452 |
|  | GSE17537 | DSS | 49 | 0.54 [0.24 - 1.23] | 0.145 |
| Eye cancer | GSE22138 | DMFS | 63 | 1.91 [1.47 - 2.49] | 0.000 |
|  | GSE22138 | DMFS | 63 | 1.84 [1.44 - 2.34] | 0.000 |
| Head and neck cancer | GSE2837 | RFS | 28 | 0.04 [0.00 - 5516.26] | 0.594 |
| Lung cancer | jacob-00182-CANDF | OS | 82 | 1.36 [0.72 - 2.58] | 0.341 |
|  | jacob-00182-CANDF | OS | 82 | 1.32 [0.83 - 2.10] | 0.243 |
|  | HARVARD-LC | OS | 84 | 0.47 [0.18 - 1.22] | 0.122 |
|  | jacob-00182-HLM | OS | 79 | 0.79 [0.50 - 1.25] | 0.324 |
|  | jacob-00182-HLM | OS | 79 | 0.90 [0.68 - 1.19] | 0.453 |
|  | jacob-00182-MSK | OS | 104 | 0.94 [0.58 - 1.52] | 0.796 |
|  | jacob-00182-MSK | OS | 104 | 0.85 [0.45 - 1.60] | 0.610 |
|  | GSE13213 | OS | 117 | 0.82 [0.55 - 1.21] | 0.322 |
|  | GSE31210 | RFS | 204 | 1.60 [0.93 - 2.75] | 0.090 |
|  | GSE31210 | OS | 204 | 1.00 [0.51 - 1.98] | 0.995 |
|  | GSE31210 | RFS | 204 | 1.19 [0.72 - 1.99] | 0.498 |
|  | GSE31210 | OS | 204 | 1.01 [0.49 - 2.10] | 0.976 |
|  | jacob-00182-UM | OS | 178 | 0.70 [0.44 - 1.10] | 0.124 |
|  | jacob-00182-UM | OS | 178 | 0.76 [0.54 - 1.07] | 0.121 |
|  | GSE3141 | OS | 111 | 1.20 [0.72 - 2.00] | 0.485 |
|  | GSE3141 | OS | 111 | 1.14 [0.77 - 1.69] | 0.521 |
|  | GSE14814 | OS | 90 | 1.03 [0.46 - 2.31] | 0.937 |
|  | GSE14814 | DSS | 90 | 0.81 [0.32 - 2.08] | 0.662 |
|  | GSE14814 | OS | 90 | 1.03 [0.61 - 1.76] | 0.908 |
|  | GSE14814 | DSS | 90 | 0.97 [0.53 - 1.79] | 0.926 |
|  | GSE4716-GPL3696 | OS | 50 | 14.28 [1.10 - 185.15] | 0.042 |
|  | GSE8894 | RFS | 138 | 0.83 [0.66 - 1.03] | 0.094 |
|  | GSE8894 | RFS | 138 | 0.92 [0.76 - 1.12] | 0.403 |
|  | GSE4573 | OS | 129 | 0.68 [0.36 - 1.26] | 0.215 |
|  | GSE4573 | OS | 129 | 0.51 [0.20 - 1.28] | 0.151 |
|  | GSE17710 | RFS | 56 | 0.90 [0.58 - 1.42] | 0.664 |
|  | GSE17710 | OS | 56 | 0.70 [0.41 - 1.20] | 0.192 |
| Ovarian cancer | GSE9891 | OS | 278 | 1.27 [1.03 - 1.58] | 0.027 |
|  | GSE9891 | OS | 278 | 1.19 [0.98 - 1.44] | 0.077 |
|  | DUKE-OC | OS | 133 | 1.13 [1.00 - 1.27] | 0.050 |
|  | DUKE-OC | OS | 133 | 1.11 [0.96 - 1.30] | 0.164 |
|  | GSE8841 | OS | 81 | 1.65 [0.93 - 2.92] | 0.086 |
|  | GSE26712 | DFS | 185 | 0.90 [0.77 - 1.05] | 0.178 |
|  | GSE26712 | OS | 185 | 0.83 [0.65 - 1.05] | 0.126 |
|  | GSE26712 | OS | 185 | 0.86 [0.72 - 1.02] | 0.084 |
|  | GSE26712 | DFS | 185 | 0.89 [0.72 - 1.11] | 0.317 |
|  | GSE17260 | OS | 110 | 0.96 [0.65 - 1.41] | 0.826 |
|  | GSE17260 | PFS | 110 | 1.28 [0.96 - 1.71] | 0.087 |
|  | GSE14764 | OS | 80 | 1.28 [0.82 - 2.01] | 0.274 |
|  | GSE14764 | OS | 80 | 1.71 [0.87 - 3.34] | 0.120 |
| Prostate cancer | GSE16560 | OS | 281 | 1.08 [0.89 - 1.30] | 0.450 |
| Skin cancer | GSE19234 | OS | 38 | 2.09 [0.77 - 5.66] | 0.148 |
|  | GSE19234 | OS | 38 | 2.06 [1.02 - 4.15] | 0.043 |
| Soft tissue cancer | GSE30929 | DRFS | 140 | 1.16 [0.72 - 1.89] | 0.542 |
|  | GSE30929 | DRFS | 140 | 1.34 [0.90 - 1.99] | 0.144 |

OS:Overall Survival. DSS:Disease Specific Survival.EFS:Event Free Survival.DMFS:Distant Metastasis Free Survival.DFS:Disease Free Survival.RFS:Relapse Free Survival.PFS:Progression Free Survival.DRFS:Distant Recurrence Free Survival.

Supplementary Table 3. Correlation analysis between PTP4A3 and related genes and markers of immune cells in TIMER.

| Description | Gene markers | KIRC | | | | KIRP | | | |
| --- | --- | --- | --- | --- | --- | --- | --- | --- | --- |
|  |  | None | | Purity | | None | | Purity | |
|  |  | Cor | *P* | Cor | *P* | Cor | *P* | Cor | *P* |
| B cell | CD19 | 0.132 | 2.19E-03 | 0.067 | 1.49E-01 | 0.134 | 2.26E-02 | 0.171 | 5.99E-03 |
|  | CD79A | 0.147 | 6.92E-04 | 0.061 | 1.93E-01 | 0.171 | 3.55E-03 | 0.209 | 7.26E-04 |
| Monocyte | CD86 | 0.019 | 6.56E-01 | -0.098 | 3.46E-02 | 0.117 | 4.69E-02 | 0.154 | 1.34E-02 |
|  | CD115(CSF1R) | 0.174 | 5.63E-05 | 0.083 | 7.59E-02 | 0.215 | 2.38E-04 | 0.272 | 9.17E-06 |
| TAM | CCL2 | 0.063 | 1.44E-01 | 0.020 | 6.71E-01 | 0.068 | 2.49E-01 | 0.089 | 1.54E-01 |
|  | CD68 | -0.052 | 2.27E-01 | -0.114 | 1.39E-02 | -0.100 | 8.93E-02 | -0.109 | 8.02E-02 |
|  | IL10 | 0.110 | 1.11E-02 | 0.017 | 7.08E-01 | 0.130 | 2.71E-02 | 0.143 | 2.18E-02 |
| M1 Macrophage | INOS(NOS2) | 0.340 | 6.30E-16 | 0.300 | 4.83E-11 | 0.265 | 4.90E-06 | 0.297 | 1.14E-06 |
|  | IRF5 | -0.158 | 2.56E-04 | -0.218 | 2.42E-06 | 0.105 | 7.29E-02 | 0.108 | 8.39E-02 |
|  | COX2(PTGS2) | 0.242 | 1.56E-08 | 0.233 | 4.03E-07 | 0.197 | 7.47E-04 | 0.225 | 2.63E-04 |
| M2 Macrophage | CD163 | 0.117 | 6.92E-03 | 0.048 | 3.05E-01 | 0.133 | 2.38E-02 | 0.151 | 1.53E-02 |
|  | VSIG4 | 0.125 | 3.94E-03 | 0.037 | 4.32E-01 | 0.173 | 3.14E-03 | 0.201 | 1.17E-03 |
|  | MS4A4A | 0.124 | 4.25E-03 | 0.028 | 5.47E-01 | 0.091 | 1.23E-01 | 0.124 | 4.68E-02 |
| Neutrophils | CD66 b(CEACAM8) | 0.030 | 4.96E-01 | 0.029 | 5.31E-01 | -0.004 | 9.41E-01 | 0.009 | 8.89E-01 |
|  | CD11b(ITGAM) | 0.099 | 2.21E-02 | 0.017 | 7.12E-01 | 0.181 | 2.00E-03 | 0.203 | 1.03E-03 |
|  | CCR7 | 0.271 | 2.10E-10 | 0.187 | 5.36E-05 | 0.027 | 6.44E-01 | 0.051 | 4.14E-01 |
| Natural killer cell | KIR2DL1 | 0.165 | 1.35E-04 | 0.159 | 5.94E-04 | 0.126 | 3.23E-02 | 0.129 | 3.90E-02 |
|  | KIR2DL3 | 0.099 | 2.22E-02 | 0.077 | 1.01E-01 | 0.108 | 6.56E-02 | 0.148 | 1.75E-02 |
|  | KIR2DL4 | 0.047 | 2.76E-01 | 0.011 | 8.18E-01 | 0.172 | 3.38E-03 | 0.189 | 2.33E-03 |
|  | KIR3DL1 | 0.129 | 2.76E-03 | 0.128 | 5.97E-03 | 0.114 | 5.33E-02 | 0.121 | 5.23E-02 |
|  | KIR3DL2 | 0.161 | 1.89E-04 | 0.145 | 1.76E-03 | 0.109 | 6.34E-02 | 0.130 | 3.64E-02 |
|  | KIR3DL3 | 0.081 | 6.23E-02 | 0.057 | 2.23E-01 | 0.079 | 1.79E-01 | 0.092 | 1.38E-01 |
|  | KIR2DS4 | 0.178 | 3.52E-05 | 0.174 | 1.71E-04 | 0.102 | 8.20E-02 | 0.108 | 8.38E-02 |
| Dendritic cell | HLA-DPB1 | 0.035 | 4.26E-01 | -0.074 | 1.12E-01 | 0.143 | 1.49E-02 | 0.192 | 1.98E-03 |
|  | HLA-DQB1 | 0.069 | 1.13E-01 | -0.021 | 6.52E-01 | 0.098 | 9.71E-02 | 0.127 | 4.22E-02 |
|  | HLA-DRA | -0.022 | 6.15E-01 | -0.133 | 4.17E-03 | 0.144 | 0.014462 | 0.189 | 2.27E-03 |
|  | HLA-DPA1 | 0.009 | 8.42E-01 | -0.111 | 1.76E-02 | 0.149 | 1.10E-02 | 0.210 | 6.99E-04 |
|  | BDCA-1(CD1C) | 0.208 | 1.20E-06 | 0.139 | 2.74E-03 | 0.185 | 1.58E-03 | 0.225 | 2.65E-04 |
|  | BDCA-4(NRP1) | 0.384 | 3.42E-20 | 0.356 | 3.51E-15 | 0.060 | 3.09E-01 | 0.087 | 1.61E-01 |
|  | CD11c(ITGAX) | 0.080 | 6.65E-02 | 0.026 | 5.73E-01 | 0.080 | 1.76E-01 | 0.106 | 8.86E-02 |
| CD8+T cell | CD8A | -0.007 | 8.78E-01 | -0.117 | 1.18E-02 | 0.103 | 8.12E-02 | 0.143 | 2.14E-02 |
|  | CD8B | -0.015 | 7.22E-01 | -0.120 | 1.01E-02 | 0.077 | 1.90E-01 | 0.109 | 7.98E-02 |
| T cell(general) | CD3D | 0.049 | 2.56E-01 | -0.076 | 1.05E-01 | 0.124 | 4.64E-02 | 0.135 | 3.02E-02 |
|  | CD3E | 0.061 | 1.56E-01 | -0.060 | 1.97E-01 | 0.069 | 2.39E-01 | 0.107 | 8.56E-02 |
|  | CD2 | 0.040 | 3.53E-01 | -0.088 | 5.88E-02 | 0.078 | 1.87E-01 | 0.127 | 4.08E-02 |
| Th1 | T-bet(TBX21) | 0.279 | 5.60E-11 | 0.234 | 3.57E-07 | 0.071 | 2.25E-01 | 0.080 | 2.03E-01 |
|  | STAT4 | 0.229 | 9.00E-08 | 0.141 | 2.36E-03 | 0.027 | 6.51E-01 | 0.068 | 2.80E-01 |
|  | STAT1 | -0.005 | 9.02E-01 | -0.107 | 2.16E-02 | 0.129 | 2.86E-02 | 0.146 | 1.89E-02 |
|  | IFN-γ(IFNG) | -0.015 | 7.22E-01 | -0.123 | 7.97E-03 | 0.111 | 5.80E-02 | 0.119 | 5.61E-02 |
|  | TNF-α(TNF) | 0.122 | 4.86E-03 | 0.059 | 2.06E-01 | 0.143 | 1.46E-02 | 0.166 | 7.55E-03 |
| Th2 | GATA3 | 0.118 | 6.48E-03 | 0.090 | 5.42E-02 | 0.198 | 6.96E-04 | 0.233 | 1.57E-04 |
|  | STAT6 | 0.138 | 1.43E-03 | 0.143 | 2.06E-03 | 0.068 | 2.51E-01 | 0.074 | 2.38E-01 |
|  | STAT5A | 0.086 | 4.61E-02 | -0.017 | 7.14E-01 | 0.231 | 7.26E-05 | 0.239 | 1.04E-04 |
|  | IL13 | 0.164 | 1.46E-04 | 0.178 | 1.20E-04 | 0.089 | 1.30E-01 | 0.056 | 3.72E-01 |
| Tfh | BCL6 | 0.249 | 5.55E-09 | 0.253 | 3.44E-08 | -0.001 | 9.85E-01 | 0.014 | 8.27E-01 |
|  | IL21 | 0.014 | 7.56E-01 | -0.015 | 7.51E-01 | 0.082 | 1.66E-01 | 0.083 | 1.84E-01 |
| Th17 | STAT3 | 0.249 | 5.84E-09 | 0.221 | 1.62E-06 | 0.197 | 7.62E-04 | 0.225 | 2.74E-04 |
|  | IL17A | 0.032 | 4.57E-01 | 0.003 | 9.53E-01 | -0.030 | 6.10E-01 | -0.033 | 5.94E-01 |
| Treg | FOXP3 | 0.153 | 4.10E-04 | 0.063 | 1.77E-01 | 0.098 | 9.42E-02 | 0.096 | 1.24E-01 |
|  | CCR8 | 0.071 | 9.99E-02 | -0.019 | 6.78E-01 | 0.159 | 6.65E-03 | 0.171 | 6.01E-03 |
|  | STAT5B | 0.176 | 4.30E-05 | 0.176 | 1.51E-04 | 0.055 | 3.53E-01 | 0.072 | 2.47E-01 |
|  | TGFβ(TGFB1) | 0.510 | 1.37E-36 | 0.502 | 8.27E-31 | 0.421 | 1.72E-12 | 0.378 | 3.75E-11 |
| T cell exhaustion | PD-1(PDCD1) | 0.006 | 8.88E-01 | -0.083 | 7.62E-02 | 0.144 | 1.39E-02 | 0.183 | 3.12E-03 |
|  | CTLA4 | 0.026 | 5.53E-01 | -0.075 | 1.06E-01 | 0.120 | 4.12E-02 | 0.131 | 3.56E-02 |
|  | LAG3 | -0.012 | 7.74E-01 | -0.108 | 2.02E-02 | 0.178 | 2.35E-03 | 0.216 | 4.85E-04 |
|  | TIM-3(HAVCR2) | -0.085 | 5.07E-02 | -0.149 | 1.32E-03 | -0.022 | 7.07E-01 | -0.017 | 7.83E-01 |
|  | GZMB | 0.215 | 5.46E-07 | 0.164 | 3.94E-04 | 0.191 | 1.12E-03 | 0.221 | 3.58E-04 |

Cor, R Value of Spearman’s correlation; None, correlation without adjustment. Purity, correlation without tumor purity.

Abbreviations: KIRC, Kidney renal clear cell cancer; KIRP, Kidney renal papillary cancer.

Supplementary Table 4. The primers for Real-time PCR.

| Gene symbol | Forward primer sequence (5'-3') | Reverse primer sequence (5'-3') |
| --- | --- | --- |
| *CD115(CSF1R)* | GCTGCCTTACAACGAGAAGTGG | CATCCTCCTTGCCCAGACCAAA |
| *CCR8* | TGGCTGTTGTCCATGCCGTGTA | TGGGATGGTAGCCATAATGGCG |
| *IL-1 alpha* | AGATGCCTGAGATACCCAAAACC | CCAAGCACACCCAGTAGTCT |
| *MMP-11* | CCGCAACCGACAGAAGAGG | ATCGCTCCATACCTTTAGGGC |
| *TGFB1* | TACCTGAACCCGTGTTGCTCTC | GTTGCTGAGGTATCGCCAGGAA |
| *GAPDH* | CATCAAGAAGGTGGTGAAGCAG | CGTCAAAGGTGGAGGAGTGG |
